# Evidence for climate-mediated range expansion of hybrid wood ants

**DOI:** 10.1016/j.isci.2026.117834

**Published:** 2026-10-15

**Authors:** Patrick Krapf, Patrick Heidbreder, Marjolein Bruijning, Patrick G. Meirmans, Sean Stankowski, Jonna Kulmuni

**Affiliations:** 1 Department of Evolutionary and Population Biology, Institute for Biodiversity and Ecosystem Dynamics, University of Amsterdam, 1098 XH Amsterdam, the Netherlands; 2 Organismal and Evolutionary Biology Research Programme, University of Helsinki, 00790 Helsinki, Finland; 3 Genetics, Evolution and Environment, University College London, London WC1E 6BT, UK; 4 Department of Ecology and Evolution, School of Life Sciences, University of Sussex, Brighton BN1 9RH, UK; 5 Tvärminne Zoological Station, University of Helsinki, 10900 Hanko, Finland

**Keywords:** hybridization, *Formica rufa* group, *Formica aquilonia*, *Formica aquilonia* × *Formica polyctena* hybrids, CTmax assay, acute heat shock, whole-genome sequencing, phenology, survival analysis, climate change

## Abstract

Climate change challenges many species. To persist, species can shift ranges, respond plastically, and adapt, which all require variation. Hybridization can quickly increase genetic variation, potentially facilitating climate adaptation; however, it is unclear how hybrids respond to climatic changes. Here, we combine whole-genome, life-history, acute heat-shock, and climatic data of 69 wood ant populations across Finland to assess whether *F. aquilonia × F. polyctena* hybrids outperform their cold-adapted parent *F. aquilonia* in warming climates. Compared to *F. aquilonia*, hybrids are active and their offspring emerge earlier in spring, and they withstand acute temperatures relevant in nature better. Further, the hybrids’ border has shifted 200 km northwards, coinciding with the expansion of climatic conditions suitable for hybrids. Our results provide evidence that hybrids have an adaptive advantage over *F. aquilonia* due to their admixed ancestry, and recent climatic changes can lead to range expansion of hybrids.

## Highlights

• *F. aquilonia × F. polyctena* hybrids are frequently found in Southern Finland
• Hybrids are active earlier, and sexual offspring emerge earlier, than those in *F. aquilonia*
• Hybrids cope well with acute heat stress due to admixed ancestry
• Hybrids have expanded ∼200 km northward over 18 years, likely due to climate warming

## Graphical abstract

**Figure:**
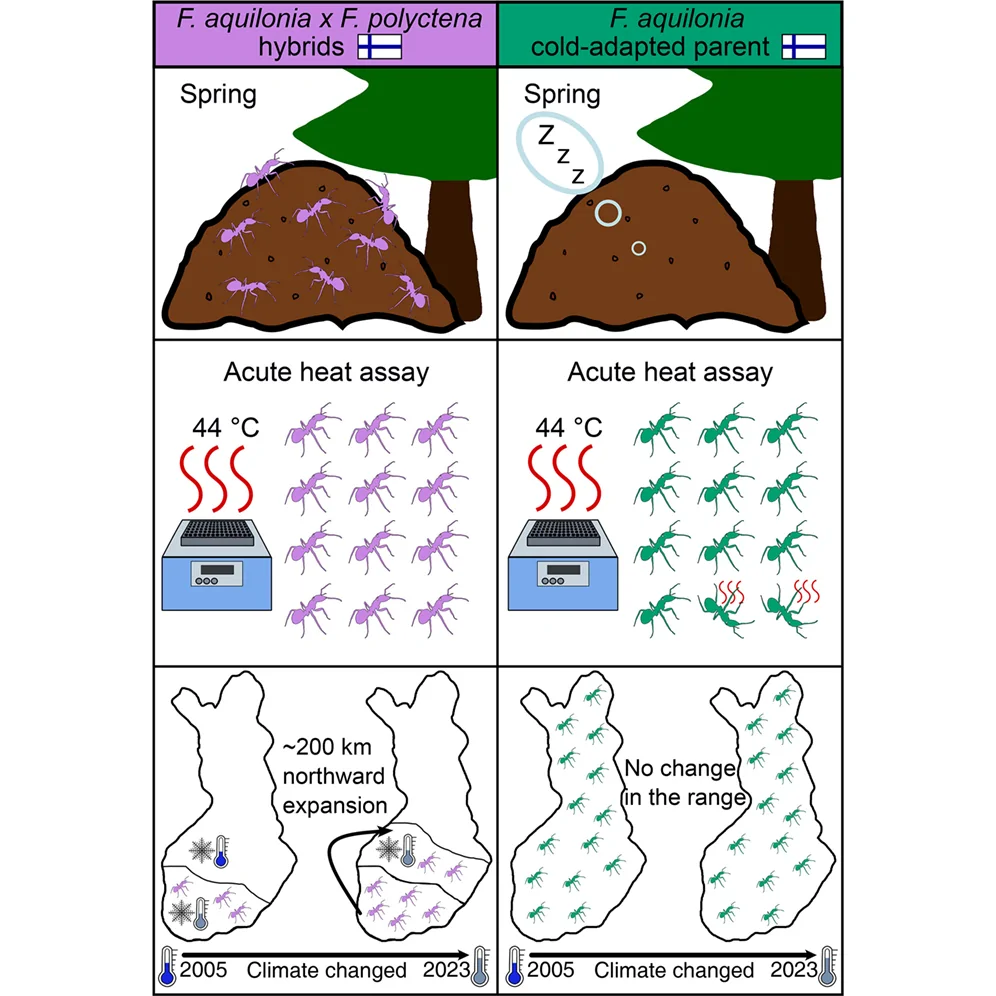

## Teaser

Global change; Biological sciences; Entomology

## Introduction

Climate change is ubiquitous and challenges nearly every species. Since 1900, average global temperatures have drastically increased,^1^ with 2023, 2024, and 2025 being the hottest years since records began (Copernicus CS3). Concurrently, the frequency of extreme events such as heat waves, droughts, and heavy rainfall has dramatically increased in the last decades.^1^^,^^2^ The effects of higher temperatures and climate change are diverse^3^^,^^4^^,^^5^ and are one of the major drivers causing global biodiversity losses.^6^^,^^7^^,^^8^ It is thus pressing and fundamental to understand and predict how species respond and persist under ongoing climate change.

Species can cope with changing climates through shifts in their habitat range, phenotypic plasticity, adaptation via genomic changes, or a combination thereof.^9^ Winged species may be able to rapidly shift ranges and find suitable habitats.^10^^,^^11^ In contrast, rapid range shifts are less likely for taxa with limited dispersal capacities, such as many plant and some animal species.^11^^,^^12^ Alternatively to shifting their ranges, species can “acclimatize” via phenotypic plasticity. It allows individuals to adjust their phenotype to different environments within an organism’s lifetime and without an underlying genomic change.^13^ However, plasticity is finite, costly, and limited by the genomic makeup of an organism, and may constrain genomic adaptation.^14^ Hence, it is expected that many species will need to rely on rapid adaptation in a fast-changing world to persist.^15^^,^^16^

Adaptation requires time and standing or novel genomic variation. While genomic variation may be present in large populations or populations with high mutation rates, many natural populations are small—often due to anthropogenic activities. As a result, they have low levels of genomic variation.^17^ Here, we define hybridization as interbreeding between different populations or species producing offspring of mixed ancestry.^18^ Hybridization has for long time been associated with deleterious consequences, such as inviability and sterility of hybrids,^19^ or hybrid breakdown in later generations of hybrids.^20^ However, in recent years this perspective has shifted: hybridization can also be beneficial and lead to introgression of beneficial alleles allowing rapid adaptation.^21^^,^^22^ Due to “mixing” genomes of two distinct lineages, it can quickly increase genomic diversity,^23^ thereby increasing the adaptive potential of populations.^16^ For example, hybrid offspring of sunflowers *Helianthus annuus* and *H. petiolaris* can persist in extreme environments, such as deserts or salt marshes, while the parents cannot.^24^ Also, hybrid rainbowfish^25^ and oak^26^ seem to be better equipped to cope with rapid climatic changes. Altogether, such elevated genomic and phenotypic variation in viable hybrids could make them better equipped to persist in rapidly changing environments.^21^^,^^22^

To date, knowledge on the extent of hybridization in natural populations is limited. Based on morphological data, the percentage of hybridizing species was estimated to be approximately 10% in animals and 25% in plants.^27^ However, these estimates predate the advent of modern genomic analyses, and true rates are likely higher as hybrids frequently remain undetected.^27^ Irrespective of the exact hybridization rates, they are expected to increase with natural and human-driven processes. For example, a recent southward move in an Atlantic puffin population led to the formation of a hybrid population in the Arctic.^28^ This is because a changing climate can cause range shifts^15^ thus increasing the likelihood that previously isolated populations will interact.^29^ Besides increasing the chances of increased contact, a changing climate can also lead to the movement of taxa, as seen in hybrid chickadees moving northwards^30^ as well as other taxa.^31^ While these examples illustrate that hybrid populations are forming and moving in response to climate change, we lack an understanding of whether hybridization also promotes adaptation to climatic changes.^22^^,^^32^

The *Formica* mound-building wood ants are a highly suitable group to study hybridization and its implication for climate change adaption. This relatively young group has diverged into up to 13 species^33^ within the last 500,000 years.^34^ Five of these occur in Finland with many of them hybridizing naturally (see the following text and work by Seifert et al.^35^^,^^36^), and hybrid populations account for a significant proportion of wood ant populations in Finland.^37^

Wood ants are keystone forest species. They are frequently found in temperate and boreal forests where they provide important ecosystem services such as re-cycling nutrients,^38^ protecting the forest against bark beetles,^39^ and supporting over 200 other invertebrate species.^38^ The species differ in their social structures, dispersal abilities, and climatic adaptation. Three of the five species found in Finland are mainly single-queened and conduct long-range dispersal (*Formica lugubris*, *F. pratensis*, and *F. rufa*), while the other two are multiple-queened and conduct short-range dispersal (*F. aquilonia* and *F. polyctena*).^35^^,^^38^ However, the social structure and dispersal abilities do not reflect climatic adaptation: *Formica aquilonia* and *F. lugubris* are cold-adapted and are found at higher latitudes in Scandinavia and higher elevations in the European Alps. In contrast, *F. polyctena*, *F. pratensis*, and *F. rufa* are warm-adapted and found at lower latitudes in Scandinavia and at lower to medium elevations in the European Alps.^40^^,^^41^ All wood ants also form characteristic and semi-stationary mounds, which can remain in the same location for multiple years.^38^ They thus need to respond to changing climates to ensure their persistence.

In Southern Finland, stable *F. aquilonia* × *F. polyctena* hybrid populations (as of now “hybrids”) are known.^21^^,^^36^^,^^37^^,^^42^ Recent simulation studies on *F. aquilonia, F. polyctena*, and these hybrid populations have shown that parental species have diverged from each other ∼523,900 to ∼533,193 years ago (∼224,000 generations) with gene flow mainly from cold-adapted *F. aquilonia* into warm-adapted *F. polyctena*.^43^ These simulations also suggest that hybridization has been ongoing ever since. We assume that in Southern Finland, hybrids have been mating within hybrid populations. Also, two contemporary hybrid populations have formed in the early 1900s^42^ and persisted ever since.^36^ However, we currently lack knowledge of whether other hybrid populations exist across Finland.

We define hybrids as individuals that display mixed ancestry between the parental species *F. aquilonia* and *F. polyctena* in the admixture analyses, as well as their intermediate spatial placement between parental species in a principal component analysis (PCA).^37^ These hybrid populations differ in some respects from their parental species. For example, hybrids are found in warmer and drier microclimates than populations of locally dominant *F. aquilonia*.^37^ Moreover, earlier results suggest that hybrids and *F. polyctena* perform significantly worse under acute cold shock than *F. aquilonia*.^44^ In contrast, hybrids withstand prolonged heat stress equally well as warm-adapted parent *F. polyctena* and significantly better than the cold-adapted parent *F. aquilonia*.^44^ However, data on acute heat shock are lacking. Also, hybrids harbor variation relevant for climate, as selection favors alleles from the warm-adapted parental species in warm springs and alleles from cold-adapted parental species in cold springs.^44^ These findings suggest that hybrid wood ants may better cope with a rapidly changing climate than parental species. However, extensive interdisciplinary analyses across space and time comparing hybrids and parents are needed to prove such broad-scale responses to a warming climate.

Here, we integrate whole-genome sequencing, life-history, acute heat stress, and climatic data on wood ant populations across Finland. This extensive and unique dataset allows us to identify *F. aquilonia* × *F. polyctena* hybrid populations across Finland and compare hybrids with co-occurring cold-adapted *F. aquilonia* populations. Specifically, we sampled 92 populations, out of which 48 and 21 populations were *F. aquilonia* and *F. aquilonia* × *F. polyctena* hybrids, respectively. We show that hybrids are frequently found in Finland, and they have a greater genomic diversity, which can lead to adaptive benefits. Hybrids are also likely to outperform *F. aquilonia* due to an earlier onset of mound activity, emergence of sexuals in the spring, and an increased tolerance to acute heat stress. Consistent with these advantages, hybrid populations have persisted over 18 years, become slightly more abundant, and expanded their range to the north. The expansion of hybrid range coincides with expansion of climatic conditions suitable for hybrids over the past 18 years. Overall, our data suggest that increasing temperatures due to climate change have facilitated a northward expansion of these hybrid wood ants. These findings highlight the major evolutionary and ecological role that hybrids can play in responding to a changing climate.

## Results and discussion

### *F. aquilonia* × *F. polyctena* wood ant hybrids are abundant in Finland

Our first goal was to determine the distribution of *F. aquilonia*, *F. polyctena*, and their hybrids across contemporary Finland. To do this, we sampled 92 wood ant populations in 2023 (‘current sampling') and used whole genome sequencing to identify species and hybrids. We found 48 *Formica aquilonia* (52% of all samples) and 21 *Formica aquilonia* × *F. polyctena* hybrid (23%) populations. In contrast to earlier morphological studies in Finland,^45^^,^^46^ we found no *F. polyctena* populations, based on whole-genome sequencing. We also detected two other *Formica* species and additional hybrids in the remaining samples with a relative frequency of <13% (Figures S1 and S2 and Tables S1 and S2). We used whole-genome sequencing for species identification, and it remains a possibility that previous studies based on morphological identification have misidentified species.

In the current dataset, we found *F. aquilonia* × *F. polyctena* hybrid populations more frequently than several other wood ant species in Finland compared to previous studies, and hybrids increased in frequency. In our dataset, 21 *F. aquilonia* × *F. polyctena* hybrid populations were found, which represents a 75% relative increase from the 12 populations observed in a previous study (‘previous sampling') using a similar number of samples (N_2023_ = 21/92 (23%); N_2005–2019_ = 12/87 (12%); Table S2;^37^). The results indicate that hybrids are persistent and one of the most abundant wood ant taxa in Finland. Further, the ratio of hybrids compared to parental *F. aquilonia* (23% vs. 52%, respectively) is high and also similar or higher compared with other hybridizing species pairs (some European ant species: 12%–31%,^47^; *Heliconius* butterflies: 7%–13%;^48^). For the remainder of this study, we focus on the comparison between *F. aquilonia* × *F. polyctena* hybrids and cold-adapted parent *F. aquilonia* across their sympatric area (i.e., area where hybrids and *F. aquilonia* co-occur), unless stated otherwise.

In the sympatric area, hybrids have a higher genomic diversity than their cold-adapted parent *F. aquilonia*. We calculated the observed heterozygosity using whole-genome data as a proxy for genomic diversity (Welch’s *t* test: H_O_ hybrids: 0.11; *F. aquilonia*: 0.08; t = −4.70, degree of freedom [df] = 23.58, *p* value < 0.001; Figure 1A). The observed heterozygosity values are similar to those of the ‘previous' sampling showing that hybrids have maintained diversity over time (H_O_ hybrids: 0.10; *F. aquilonia*: 0.08;^37^). We also calculated nucleotide diversity over 10 kb windows, which was higher in hybrids (hybrids: 0.0017; *F. aquilonia*: 0.0014; Welch’s *t* test = −47.57, df = 39927, *p* value < 0.001). Such a higher genomic diversity observed in hybrids compared to *F. aquilonia* could contribute to faster adaptation to climatic changes.^21^^,^^24^

**Figure 1:**
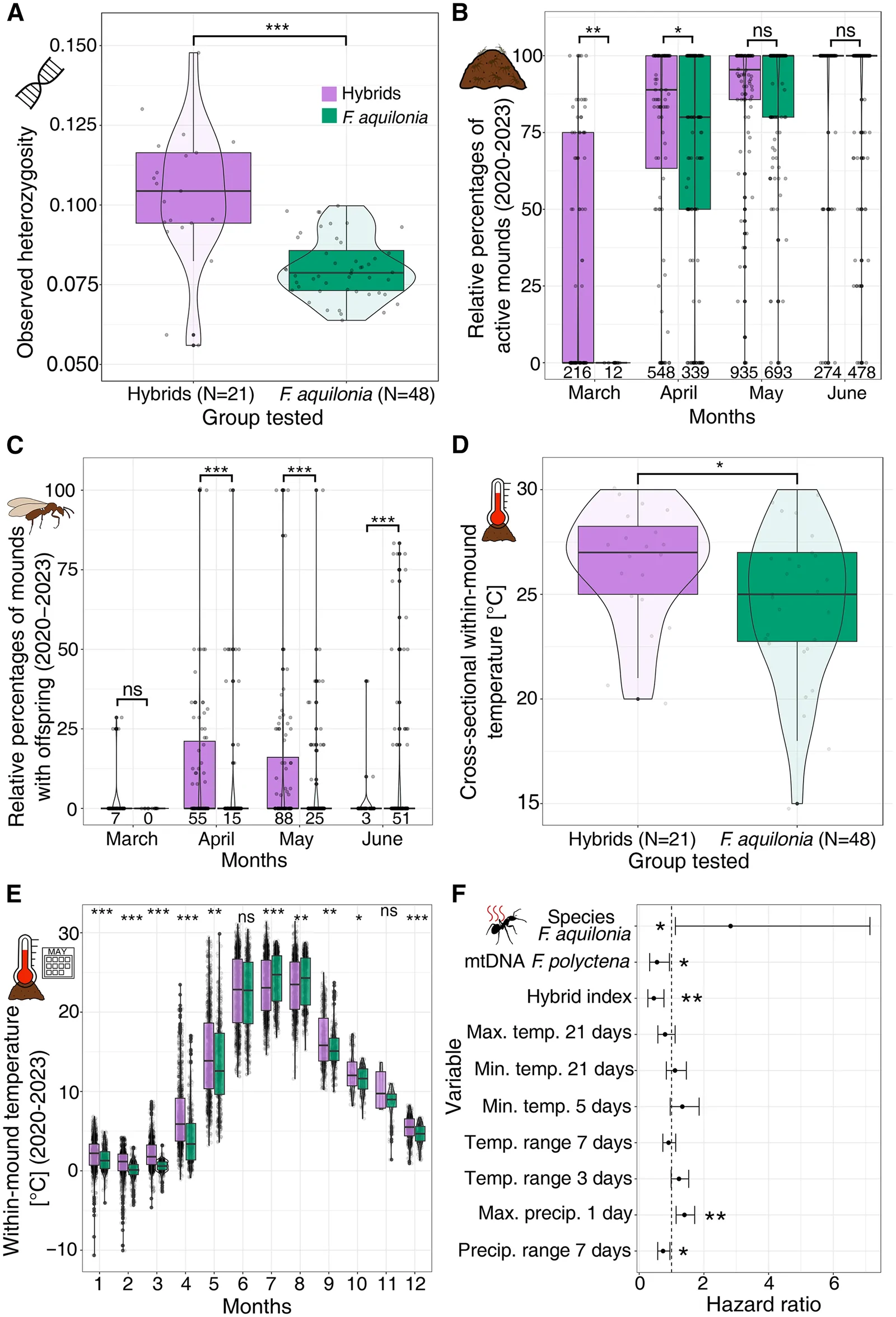
Recorded traits and characteristics for *F. aquilonia × F. polyctena* hybrids (purple) and *F. aquilonia* (green) populations (A) Observed heterozygosity including a mean comparison (Student’s *t* test) between hybrids and *F. aquilonia*. (B) Relative percentages of active mounds in Southern Finland recorded between March and June for the years 2020–2023 for hybrids and *F. aquilonia* calculated using a proportion test and corrected for multiple testing. (C) Relative percentages of mounds with offspring in Southern Finland observed from 2020 to 2023 calculated using a proportion test and corrected for multiple testing. The numbers below the boxplots in (B) represent the numbers of mounds checked for (B) and (C). The numbers below the boxplots in (B) represent the numbers of mounds with offspring observed. (D) Within-mound temperature measured at the top of the mound recorded during the sampling in the field in the year 2023 calculated using a Student’s *t* test. (E) Within-mound temperature measured from HOBO loggers between 2020–2023 between hybrids and *F. aquilonia* (HOBO data points for hybrid mounds for each month over the three years = 837, 756, 837, 870, 961, 930, 961, 793, 383, 124, 8, and 372; HOBO data points for *F. aquilonia* mounds each month over the three years = 341, 336, 372, 360, 480, 480, 496, 440, 293, 176, 76, and 186). The data were analyzed using a Mann-Whitney U-test and corrected for multiple testing. (F) Expected hazard ratios to fall into heat coma presented for the species identity, mtDNA origin, hybrid index, and several weather variables prior to sampling shown using a forest plot. The dots represent the estimated hazard ratios and the whiskers represent their 95% confidence intervals. Note, in (A), (D), and (E), the interquartile range (25th-75th percentiles) is represented by the box, while the median is represented by the line within the box. Most extreme observations within the 1.5 times the interquartile range below and above the percentiles are presented with whiskers. Observations beyond the whiskers are presented as dots. The width in the violin plots represents the density of the data at specific values, while the light gray points represent the observed values. In all panels, significant comparisons are represented with asterisks, where ∗ denotes *p* value < 0.05, ∗∗ *p* value < 0.01, ∗∗∗ *p* value < 0.001, and ns, not significant.

We further calculated the hybrid index and identified the mtDNA origin which showed that all *F. aquilonia* × *F. polyctena* populations are later-generation hybrids. The hybrid index ranged from 0.32 to 0.66 (i.e., scale 0 to 1, where 0 means only *F. polyctena* alleles are present and 1 means only *F. aquilonia* alleles; 359 ancestry informative markers; Figure S3A;^42^^,^^49^) and indicates limited backcrossing. In nine out of 21 hybrid populations (43%), mtDNA originated from cold-adapted *F. aquilonia* mothers, and in 12 (57%) from warm-adapted *F. polyctena* mothers, their frequencies thus being indistinguishable from 50:50. This result differs starkly from the ‘previous' sampling, which identified ten *F. polyctena* maternal lines (83%) but only two *F. aquilonia* maternal lines in hybrids (17%^37^). For the hybrid samples, we also calculated pairwise mtDNA differences between the maternal lineages originating from both parental species. The mutations ranged from 76 to 86 mutations (Figure S3B) which corresponds to previous results.^37^

### Hybrids differ in several characteristics from the cold-adapted parent *F. aquilonia*

Collected long-term data on the activity of ants and the emergence of sexual offspring show that hybrids are active earlier in the season, and their offspring emerge earlier and in greater numbers than *F. aquilonia* offspring. These data were recorded in three hybrid and three *F. aquilonia* populations from March until June from 2020 to 2023. Hybrid mounds were active earlier in spring than *F. aquilonia* mounds (Figure 1B, relative proportions calculated over all years; proportion test; March, X^2^ = 10.64, df = 1, *p* value < 0.001; April, X^2^ = 11.56, df = 1, *p* value < 0.001; May, X^2^ = 3.18, df = 1, *p* value = 0.075; June, X^2^ = 1.71, df = 1, *p* value = 0.191; corrected for multiple testing). Also, offspring from hybrid mounds emerged already in March and were significantly more frequently found in April and May, compared with *F. aquilonia* mounds, where significantly more offspring emerged in June (Figure 1C; relative percentages calculated for observed mounds; two-sample test for equality of proportions with continuity correction; March, X^2^ < 0.001, df = 1, *p* value = 1.000; April, X^2^ = 8.32, df = 1, *p* value = 0.004; May, X^2^ = 19.87, df = 1, *p* value < 0.001; June X^2^ = 22.54, df = 1, *p* value < 0.001).

Further, cross-sectional and long-term data demonstrate that the within-mound temperature differs between hybrids and *F. aquilonia*. The within-mound temperature was warmer in the 21 hybrid mounds than in the 48 *F. aquilonia* mounds (measured from the top of the mound; hybrids mean = 26.38°C; *F. aquilonia* mean = 24.45°C, Figure 1D; Welch’s *t* test, t = −2.98, *p* value = 0.019). Similarly, the long-term within-mound temperature measured from 2020 to 2023 (using HOBO data loggers) from six populations was significantly higher in hybrid mounds than in *F. aquilonia* mounds for almost the entire year, except for June and November (Figure 1E and Table S3). In contrast, within-mound temperature was significantly warmer in *F. aquilonia* mounds in July and August than in hybrid mounds.

The observed differences in spring activity are likely due to differences in the timing of diapause termination. Endogenous mechanisms are mainly controlling diapause length in wood ants,^50^^,^^51^ but diapause is also affected by local adaption to climates.^50^ In contrast, temperature or photoperiod is expected to have little effect on diapause.^50^^,^^51^ Cold-adapted *F.aquilonia* is likely genetically encoded to long winters^41^ but also adapted to them in Finland thus having a longer diapause than, for example, warm-adapted wood ant species. Further, with climate change, the frequency of warmer winters is expected to increase, which may negatively impact *F. aquilonia* survival in future^52^: if winters are warm, Finnish *F. aquilonia* workers wake up during diapause thus using up fat reserves, which can lead to a reduced worker survival in subsequent spring.^52^ Hybrids, in contrast, with their genetic contribution of Southern European *F. polyctena*, may be encoded and adapted to shorter winters and having a shorter diapause, as observed here. For example, in the Netherlands, *F. polyctena* mounds can already be active at the end of February (Krapf et al., unpubl.).

A shorter diapause in hybrids may represent a fitness advantage over *F. aquilonia*. A shorter diapause, and thus earlier activity, allows hybrids to start foraging earlier in the season and to take advantage of longer spring and summer seasons. It also enables the earlier emergence of sexual offspring. In turn, hybrids may have a competitive and fitness advantage in obtaining nest sites and food as well over *F. aquilonia* and also other wood ants in Finland. Further, the within-mound temperature in wood ants is influenced by workers that produce warmth with their metabolic heat^53^ as well as due to their associated microorganisms.^54^ During the season, workers also continuously regulate the nest temperature by their metabolism, partial removal of mound material to reduce wall thickness, and movement of queens and brood inside the mound.^55^ Hybrids’ higher within-mound temperature, especially in the spring, could be an effect of differing worker behaviors, associated microorganisms or myrmecophiles, and nest construction techniques. It could further be coupled with the endogenous control of the diapause and contribute to an earlier activity in spring contributing to a competitive advantage of hybrids over *F. aquilonia*.

### Hybrids perform better under acute heat stress

Given that hybrids’ mounds are warmer, and mound surface temperature can reach up to 66°C and higher (Krapf et al., unpubl.), we compared the performance of individual hybrid and *F. aquilonia* ants in experimental heat stress assays. We tested 205 ants across 18 populations in a critical thermal maximum assay (CT_max_^56^). In brief, we transferred ants to a 1.5 mL Eppendorf tube sealed with cotton and stored the tubes in a block heater (Eppendorf ThermoMix, Hamburg, Germany). We then increased the temperature from 40°C to 49°C over 20 min (Figure S4A inset) and recorded ants’ status (active or knocked down) every minute, thus simulating semi-natural hot conditions.

We found that species identity, mtDNA origin, hybrid index, and weather characteristics prior to sampling affect the survival of ants in a CT_max_ assay. We conducted a Cox proportional hazard model to integrate heat data with species identity, parental mtDNA status, hybrid index, and weather characteristics prior to sampling (e.g., minima or maxima for temperature and precipitation; overall model significance: log-rank test = 29.39, df = 10.14; *p* value = 0.001). We used *F.aquilonia* as reference and found that it has 2.8-fold higher estimated hazard to fall into heat coma earlier compared with hybrids (Figure 1F and Table S4). In contrast, hybrid ants with *F. polyctena* mtDNA and a higher hybrid index had a 45% and 55% lower estimated hazard, respectively, to fall into heat coma compared to ants with an *F. aquilonia* mtDNA or lower hybrid index. The precipitation one day prior to sampling increased the hazard of falling into heat coma earlier by 40% in hybrids and *F. aquilonia*, while an increase in the range of precipitation seven days prior to sampling reduces the hazard of falling into heat coma earlier by 26%. Further, hybrids withstand acute heat significantly better than *F. aquilonia* (44.0°C; X^2^ = 7.18, df = 1 *p* value = 0.004; asterisk in Figure S4B and Table S5). This temperature is also close to wood ants’ thermal maximum at ∼45°C.

Within hybrids, we found no difference between warm- or cold-adapted maternal mtDNA origin or the hybrid index (as a proxy for the contribution of warm-adapted alleles) in shaping acute heat tolerance. Hybrids with mtDNA of warm-adapted *F. polyctena* fell into heat coma at a similar temperature than hybrids with mtDNA of cold-adapted *F. aquilonia* (*F. polyctena*-mtDNA hybrids: 45.6°C; *F. aquilonia*-mtDNA hybrids: 45.5°C; one-sided Mann-Whitney-U-test, W = 856.0, *p* value = 0.341; Figure S4C). Within hybrids, ants also withstand acute heat similarly well over the entire CT_max_ assay regardless of the mtDNA origin (Kaplan-Meier survival analysis, *p* value = 0.40; Figure S4D). Also, hybrid index, as a proxy for a higher genomic contribution from warm-adapted *F. polyctena* did not influence hybrids’ performance under higher temperatures (linear regression, r^2^ = 0.01, F_1, 87_ = 1.901, *p* = 0.171, Figure S4E).

Our results demonstrate that hybrids perform better under acute heat stress than cold-adapted parent *F. aquilonia*. Our approach reflects naturally measured high temperatures in summer, where mound surface temperatures can reach over 66°C. Previous results with a limited number of populations hinted that hybrids perform better under long-term heat exposure than *F. aquilonia*,^44^ but knowledge on acute heat exposure for populations across latitudes was lacking. Here, we significantly increased the sample size (from 3 populations each to N_hybrids_ = 21; N_*F. aquilonia*_ = 29) as well as geographic area of where the workers originated (from 86 to 460 km maximum linear distance between the samples).

We speculate that the hybrids’ mixed ancestry as well as the mtDNA of the warm-adapted parent *F. polyctena*, likely gives hybrids an adaptive advantage over *F. aquilonia* in a warming climate. This is in line with recent results demonstrating that hybrid lineages can better cope with increasing temperatures than their parental species: for example, in the hybrid annual wildflower *Clarkia pulchella*, gene flow from a warm-adapted species had a positive effect on fitness.^57^ Under warm conditions, F1 hybrid poplar trees survived better than parental species in a common-garden experiment.^26^ Similarly, hybrid rainbowfish are expected to have reduced vulnerability to climate changes.^25^ Future studies should address underlying mechanistic links between heat tolerance and performance of hybrid lineages.

Notably, also recent weather changes can affect survival under acute heat conditions. Our results indicated that changes in precipitation prior to sampling can increase or decrease ants’ performance. This is in line with other studies showing that heat tolerance can be affected by desiccation and starvation in *Drosophila melanogaster*^58^ as well as nutrition in ants.^59^ A hot and possibly drier climate can affect foraging behaviors in ants^60^ and thus lead to different performance in heat tolerance assays.

### Hybrids have persisted and expanded 200 km northwards

To assess species and hybrid persistence over a total of 18 years, we compared the “current” data sampled in 2023 to 44 populations from the ‘previous' sampling. The ‘previous' sampling included mainly samples collected between 2005–2008, with some additional populations sampled until 2019 that were already identified in Satokangas et al.^37^ We revisited the same mounds and found the same species or hybrids in 32 populations (73%) and species’ turnover in the other 12 populations (27%; Table S6). These revisited sites included ten hybrid populations, and we found that hybrids persisted in seven (70%) populations, some of these since 2005. Previous studies have indicated two isolated hybrid populations in Southern Finland have persisted for at least 19 years.^36^ One of these is estimated to have formed independently 13 to 47 generations ago, roughly at the beginning of 1900.^42^ Here, we show, for the first time, that other hybrid populations in Southern and Central Finland have also persisted, at least 18 years (∼7 generations, assuming a generation time of 2.5 years).

The hybrid border has expanded northwards by ∼200 km in a timeframe of 18 years (Figure 2A). Data from the current sampling (i.e., collected in 2023) demonstrated that it was 1.6 times more likely to find hybrid populations at higher latitudes compared to the ‘previous' sampling when using *F. aquilonia* as a baseline (multinomial logistic regression; corrected Akaike Information Criterion for small sample sizes [AICc] = 238.30, Nagelkerke pseudo-r^2^ = 0.38, odds ratio z value = −4.39, *p* value < 0.001; delta AICc intercept-only model: 35.58; Figure S4F). In contrast, we found no evidence that the range of either parental species or other warm-adapted species has significantly changed (*F. aquilonia* no change; *F. pratensis* ∼145 m northwards shift, *F. rufa* ∼200 km southward shift; odds ratio z value = −0.28, *p* value = 0.078; Figure 2A). This indicates that the significant northwards shift is specific to hybrids. Further, this northward expansion is remarkable for such semi-stationary insects, as insects are expected to roughly move on average 12–15 km per decade.^10^^,^^11^

**Figure 2:**
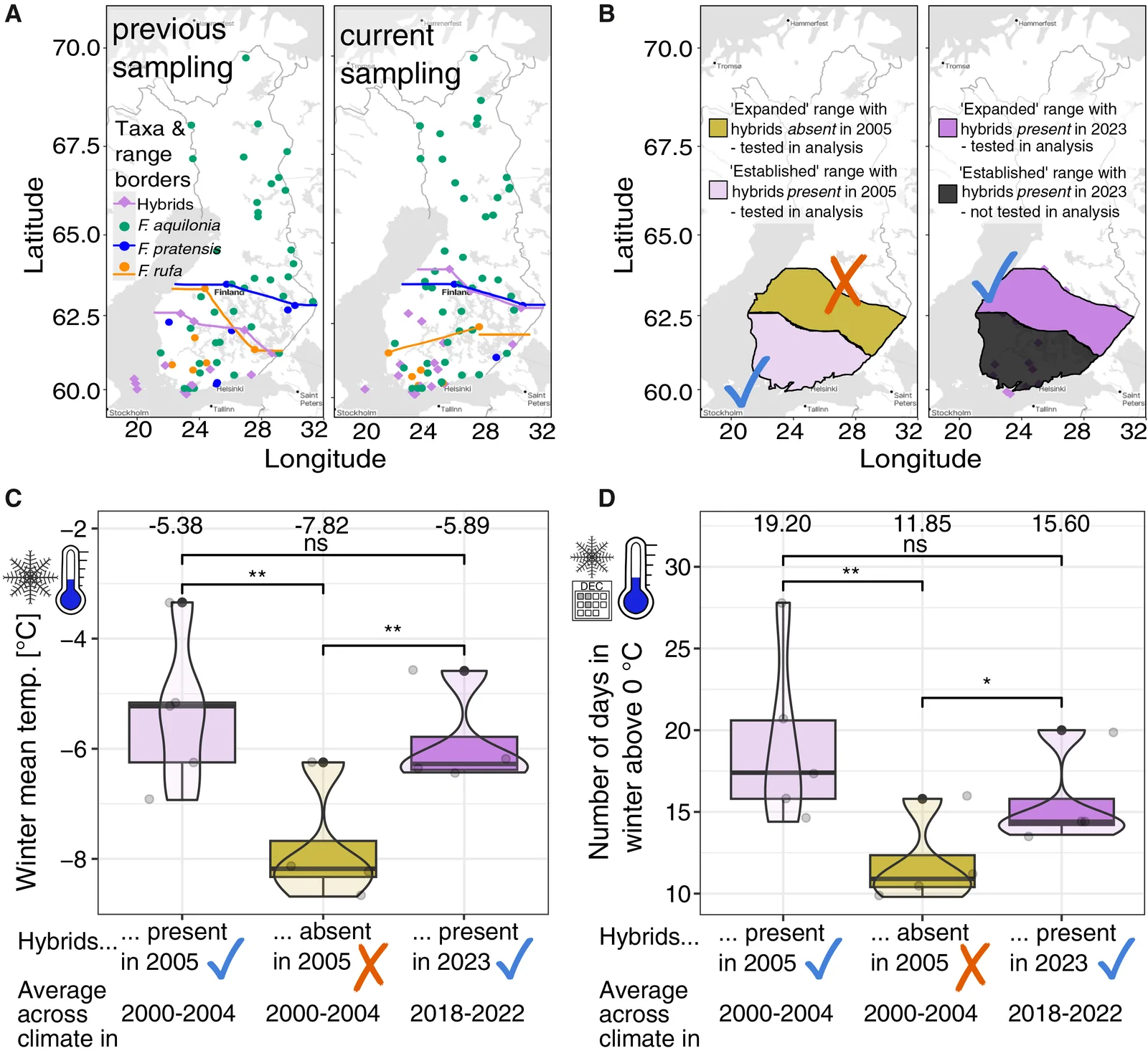
Sampling map and comparison of 5-year climatic averages of hybrid areas (A) ‘Current' and ‘previous' sampling map including taxa assignment based on whole-genome sequence data to identify wood ant species and hybrids. The ‘previous' sampling map was modified from Satokangas et al.^37^ The ‘current' sampling originates from this dataset. The lines represent species. (B) Map displaying the hybrid range and ‘expanded' range where hybrids were found and which were used for the temporal comparison. (C and D) 5-year average of climatic variables was calculated backward from the sampling year 2005 (period 1999–2004) and 2023 (period 2018–2022), which are shown below the boxplots (“average across climate in”) as well as the year when hybrid populations were collected. (C) Winter mean temperature and (D) number of days in winter above 0°C (warm winter days). “Winter” is defined as the diapause period between December to February. Values above the boxplots represent mean values. Asterisks represent bootstrapped *p* values between hybrid areas at higher and lower latitudes and over time (Table S7) with ∗ <0.05, ∗∗ <0.01, and ∗∗∗<0.001. N hybrid range in 2005 = 5. N “expanded” range in 2005 = 4. N expanded range in 2023 = 4. Note, the interquartile range (25th-75th percentiles) is represented by the box, while the median is represented by the line within the box. Most extreme observations within the 1.5 times the interquartile range below and above the percentiles are presented with whiskers. Observations beyond the whiskers are presented as dots. The width in the violin plots represents the density of the data at specific values, while the light gray points represent the observed values.

### The northward expansion of the hybrids coincides with expansion of climatic conditions suitable for hybrids

If climatic changes have allowed a northward expansion of hybrids, we expect that the climate has changed in a way that is favorable for hybrids. Specifically, we expect that the climatic conditions in the ‘expanded' range in 2023 should be similar compared to the conditions in the ‘established' range in 2005 (Figure 2B, light and dark purple areas). In contrast, the climatic conditions in the expanded range in 2005 should be unfavorable and differ from the climate in the established range in 2005 (Figure 2B; yellow vs. purple areas). To test these predictions, we calculated 5-year averages of six climatic variables (i.e., winter mean temperature, spring mean temperature, winter mean precipitation, spring mean precipitation, winter number of days above 0°C and spring number of days below 0°C), thus also accounting for yearly environmental fluctuations. Note, we used the terms “2023” and “2005” for simplicity when referring to the five-year averages from 2018 to 2022 and 2000 to 2004. Here, we only used hybrid populations, assuming that hybrid populations could follow these environmental changes and that these local climates are suitable for hybrids.

Following our expectations, the winter in 2023 (average across climate in 2018–2022) in the expanded range at higher latitudes (with hybrids present) was similarly warm and had a similar number of days above 0°C as the winter in 2005 (average across climate in 2000–2004) in the established range at lower latitudes (Figures 2C and 2D and Table S7). At the same time, and also in line with our expectations, winter in 2005 (average across climate in 2000–2004) in the expanded range (with hybrids absent) was colder and thus likely not suitable for hybrids. However, not all aspects of the climate in the expanded range have changed in such a way that matches the hybrid niche. Specifically, in spring 2023 (average across climate in 2018–2022), temperatures in the expanded range were still lower, there were more frost days below 0°C, and there was more precipitation than in the spring in 2005 (average across climate in 2000–2004) in the hybrid range (Figures S5A–S5D and Table S7).

We also assessed whether the climate has changed as a whole across both hybrid and *F. aquilonia* populations. For this, we compared the same six environmental variables described above within and between hybrid and *F. aquilonia* populations, and between previous and current sampling. The climate has changed similarly in both hybrid and *F. aquilonia* populations and followed the predicted climatic changes, with both warmer and wetter winters and springs. Notably, these climatic changes were more pronounced in hybrid populations than in *F. aquilonia* populations (Figures S6A–S6E and Table S8). These results suggest that both hybrids and *F. aquilonia* will need to cope with rapid changes as climate warming continues.

From these results, we can conclude that the climate appears to have become more favorable for hybrids at higher latitudes in Finland, but not in all respects. The increased winter temperatures follow our expectation and indicate that warmer winters may be more important for hybrids to expand northwards and persist than cold spells in spring, and that cold spells do not limit their persistence at higher latitudes. This rationale is supported by a study on *Aphaenogaster* ants demonstrating that increasing minimum temperatures along an elevation gradient in North America allowed the ant *A. rudis* to move upward in elevation.^61^ Similarly, increasing minimum winter temperatures are associated with the northward expansion of a hybrid zone in chickadees.^30^ In contrast, hybrid chickadees’ southward expansion seems to be hampered by biotic interactions between hybrids and parental species.^30^

In general, warming temperatures under climate change can allow organisms to expand to new ranges.^11^ Such climatic changes have also been causally linked to hybrid zone movements across multiple taxa.^31^ However, besides temperature, also other factors such as precipitation or foraging strategy can also promote range shifts.^62^ In Finland, the Baltic Sea may have also contributed to the persistence and expansion of hybrids. It allows for mild winters on the Finnish coastal area, which had, for example, also a positive effect on the number of bird species in Southern Finland in the last decade.^63^

While we have tested acute heat shock and compared maximum temperatures in winter and spring, also minimum temperatures can affect the expansion of ants.^64^ We have focused on CT_max_ rather than CT_min_ because hybrids were found to be less cold resistant than parental species *F. aquilonia*.^44^ Further, because climate is warming^1^ and CT_max_ is less plastic than CT_min_,^65^ we focused on CT_max_ but acknowledge that testing CT_min_ across multiple populations and along latitudes may be interesting in future studies. Although hybrids are vulnerable to cold winter temperatures,^44^ we suggest that milder winters^63^ due to recent climatic change^1^ favored hybrids’ northward expansion and persistence. Such warmer winters may enable hybrids to become active and produce sexual offspring earlier in spring than *F. aquilonia* also at higher latitudes or elevation. In turn, this can give hybrids an adaptive advantage in establishing territories and foraging areas, as well as expanding to new habitats. Granted, warmer winters are not a guarantee for success, and it may well be that some hybrid populations expanded northwards but did not persist due to colder winters.

### Smaller mound volume and less divergent mtDNA at higher latitudes are consistent a recent northward expansion of hybrid populations

If hybrid populations have initially formed in Southern Finland and have recently expanded northwards, or if the northward expansion is due to new hybridization events, we expect hybrid mounds at higher latitudes to be smaller (as ants increase mounds volume over time). We also expect hybrid mtDNA in the expanded range to be less diverse than at lower latitudes, since recently expanded hybrids had less time to accumulate mutations.

We found that hybrid mounds are significantly smaller at higher latitudes compared to lower latitudes (hybrid means: 9085.7 in the South and 5235.4 in the North; Welch’s *t* test, t = 1.96, df = 17.33, *p* value = 0.033; linear regression: r^2^ = 0.02, estimate = −1009.40, F-statistic = 1.32, df = 1 and 18, t value = −1.15, *p* value = 0.265; Figure S7A). Conversely, *F. aquilonia* mounds show a trend toward being larger at higher latitudes, though this difference is marginally not-significant (*F. aquilonia* means: 8191.9 in the South and 12112.3 in the North; Mann-Whitney-U-test, W = 196, *p* value = 0.057; linear regression: r^2^ = 0.08, estimate = 875.10, F-statistic = 4.77, df = 1 and 45, t value = 2.19, *p* value = 0.034). Notably, hybrid and *F. aquilonia* mounds do not differ significantly in their size regardless of whether they are found in the sympatric range or across entire Finland (Figures S7C and S7D).

Further, we also found that mtDNA of hybrids is less diverse at higher latitudes than at lower latitudes (i.e., fewer pairwise mutations in the mtDNA within the taxa; linear regression, hybrids: r^2^ = 0.01, estimate = −0.76, F-statistic = 4.19, df = 1 and 220, t value = −2.04, *p* value = 0.042, Figure S7B). In *F. aquilonia*, we found the opposite trend with a higher divergence at higher latitudes (linear regression; *F. aquilonia*: r^2^ = 0.04, estimate = 0.48, F-statistic = 104.60, df = 1 and 2254, t value = 10.23, *p* value < 0.001).

We emphasize that the r^2^ values of the two above-presented analyses are relatively small and thus only explain a small fraction of the variation observed. Nevertheless, the result is consistent with the idea that hybrids have recently expanded northwards. The reduced hybrid mound sizes at higher latitude and the lower number of mtDNA mutations in hybrids may both indicate that they started building mounds at higher latitudes more recently. Further, the higher number of mtDNA mutations in *F. aquilonia* at higher latitudes may be a signal of lower population densities in Southern Finland marking the Southern range edge of *F. aquilonia.*

Previous work in these hybrids hinted that selection favors alleles from the cold-adapted parent in cold springs and alleles from the warm-adapted parent in warm springs.^44^ If such a signal is strong, hybrids may have a higher allele frequency of cold-adapted *F. aquilonia* alleles or *F. aquilonia* mtDNA origin at higher latitudes. However, both the hybrid index and mtDNA origin were not correlated with latitude (hybrid index linear regression, r^2^ = −0.02, estimate = −0.01, F-statistic = 0.36, df = 1 and 40, t value = −0.50, *p* value = 0.622, Figure S7E; mtDNA origin ANOVA, F-value = 0.979, df = 1 and 18, *p* value = 0.336, Figure S7F). Nevertheless, we acknowledge that certain genomic regions and/or loci involved in the northward expansion and adaptation to cooler regions may not have been detected by simply looking at the ancestry proportion of the hybrid genomes.

While we present evidence for a climate-mediated range expansion of hybrid wood ants, it remains unclear how hybrids have expanded northwards. It could be that hybrids have initially formed in Southern Finland and then expanded northwards or if the northward expansion is due to new hybridization events. Below, we speculate what may have contributed to the hybrids’ expansion.

Hybrids seem to have similar polydomous colony structures as both parental species and thus probably also similar dispersal strategies. Both parents are poor at long-range dispersal via flights^66^ and new mounds are usually established by budding (i.e., queens and workers found a new mound a few meters next to the original mound^67^). Budding thus allows a slow but continuous northward expansion but does not explain the recent 200 km expansion. However, males from both parental species disperse by flight,^68^ which could explain the northward expansion in hybrids—assuming that hybrid males show the same dispersal behavior. If this were true, we would expect to find backcrosses at higher latitudes, which we did not observe, making this scenario less plausible. Alternatively, hybrid populations may form via novel hybridization events, but since *F. polyctena* is mainly absent from Finland (at least based on the genomic data presented here and in^37^), this also seems unlikely. It may be that a sparse number of *F. polyctena* populations are present in Finland, which we have not found during our studies. Such sparse *F. polyctena* populations could allow new hybridization events. Alternatively, it may also be that hybrids differ in their dispersal strategy from their parental species, and that both hybrids’ queens and males disperse by flight. Also, human-assisted dispersal of queens, which has been shown in a moving hybrid zone in *Solenopsis* ants^69^ may lead to the establishment of hybrid populations at higher latitudes. These scenarios need to be addressed in future studies.

### Conclusions and broader implications

We combine whole-genome data of wood ants sampled in 2023 across Finland, with observational, experimental, cross-sectional, and long-term environmental, as well as climatic data. Altogether, we found that *F. aquilonia* × *F. polyctena* hybrids have a higher genomic diversity than its cold-adapted parent *F. aquilonia*, hybrids are active with sexual offspring emerging earlier in the spring than in *F. aquilonia*. Hybrids also have an increased tolerance to acute heat stress due to their mixed ancestry and mtDNA origin of the warm-adapted parent. Hybrids are thus likely able to take advantage of longer spring and summer seasons and continued foraging capabilities during hot summer days, suggesting a higher adaptive potential. Further, hybrids have persisted and expanded approximately 200 km northwards in the last 18 years; and this northward expansion of the hybrids coincides with extension of winter conditions suitable for hybrids. The persistence and northward expansion of hybrids at higher latitudes are probably the result of both climate change and the hybrids’ genomic composition. Future studies should investigate if the high hybrid prevalence, persistence, and expansion are also observed across other geographic regions and across the tree of life.

With ongoing climate change,^1^ hybridization is expected to increase in frequency^15^ due to an increase in secondary contact of species and/or populations. Our results suggest that hybrid wood ants with limited dispersal abilities may have competitive advantages over resident species in the face of changing climates, similar as other organisms such as hybrid sunflowers.^24^ Hybrids are also able to track rapid changes in climate, which allows them to expand their distribution. They can thus be well-equipped to respond to or withstand climatic changes—possibly better than their parental species—as seen with these hybrid ants and in other hybrid organisms such as in oaks,^26^ birds,^30^ and fish.^25^ Further, wood ants as keystone species of boreal forests and support a wealth of biodiversity.^38^ Coupled with their abundance, wood ant hybrids play a major evolutionary and ecological role in the forest ecosystem. We thus argue that hybrids should also be considered in conservation planning,^70^ especially if they have a higher adaptive potential than parental species.

### Limitations of the study

One limitation of the study is that the r^2^ values of the linear regression correlating mound sizes and divergent mtDNA with latitude were relatively small. They thus only explain a small part of the variation observed, possibly indicating that hybrid mounds at higher latitudes are younger. Additionally, we did not test or check which underlying potential mechanisms may contribute to hybrids’ better performance under heat stress. Lastly, with the data at hand, we were able to only speculate about how hybrids may have expanded northwards, but could not test any of the theories mentioned.

## Resource availability

### Lead contact

Requests for further information and resources should be directed to and will be fulfilled by the lead contact, Patrick Krapf (p.kapf@uva.nl).

### Materials availability

This study did not generate new unique reagents.

### Data and code availability

• Raw whole-genome sequencing data have been deposited at NCBI project PRJNA1453165 and are publicly available (https://www.ncbi.nlm.nih.gov/bioproject/PRJNA1453165).
• Whole-genome and mtDNA VCF files and climate data have been deposited at Zenodo at https://zenodo.org/records/19563998, and are publicly available. Additional code for whole-genome analysis is publicly available under https://github.com/KraPat/WoodAnt_Species_and_Hybrid_Assignment.
• Any additional information required to reanalyze the data reported in this paper is available in the supplemental information.

## Acknowledgments

We thank Xavier Arnan for helpful feedback during the CT_max_ pilot studies, Benedikt Mader for help during field work, Peter Kuperus and Melanie Humphry for help during lab work, Anna Karrellos and Marit Kuijt for help with sample handling, as well as Marit Kuijt, Noora Poikela, Beatriz Portinha, and Ina Satokangas for helpful discussions during data analysis and writing. We wish to acknowledge CSC – IT Center for Science, Finland, for computational resources. The maps were created using Stadia Maps (stadiamaps.com), Stamen design (stamen.com), and OpenStreetMap (openstreetmap.org/copyright). This work was funded by the Suomen Akatemia grant no. 346805, 324485, and 353156 awarded to J.K., the University of Helsinki LUOVA Doctoral Program funded Doctoral researcher position awardee to P.H., and the European Union’s Horizon Europe program under Marie Skłodowska-Curie Actions (MSCA) - Postdoctoral fellowship grant agreement no. 101204375 awarded to P.K. The climate data used were downloaded from the Finnish Meteorological Institute, FMI (under https://paituli.csc.fi/home.html). We further thank anonymous reviewers for their feedback on an earlier version of the manuscript.

## Author contributions

Conceptualization, P.K. and J.K; methodology, P.K., M.B., P.G.M., S.S., and J.K.; software, P.K., P.H., M.B., P.G.M., S.S., and J.K.; validation, P.K.; formal analysis, P.K.; investigation, P.K. and P.H.; resources, J.K.; data curation, P.K. and P.H.; writing – original draft, P.K. and J.K.; writing – review and editing, P.K., P.H., M.B., P.G.M., S.S., and J.K.; visualization, P.K. and J.K.; supervision, J.K.; project administration, P.K. and J.K.; funding acquisition, J.K.

## Declaration of interests

The authors declare no competing interests.

## Declaration of generative AI and AI-assisted technologies in the manuscript preparation process

During the preparation of this work, the author used ChatGPT in order to help annotating R code. After using this tool or service, the author reviewed and edited the content as needed and take full responsibility for the content of the publication.

## STAR★Methods

### Key resources table

**Table:** 

REAGENT or RESOURCE	SOURCE	IDENTIFIER
**Biological samples**		

*Formica aquilonia*	Personal collection	ITIS: 576684
*Formica aquilonia×F. polyctena* hybrids	Personal collection	ITIS: na

**Chemicals, peptides, and recombinant proteins**		

DNA extraction for whole-genome sequencing	Qiagen	DNeasy Blood and Tissue Kit
DNA extraction for whole-genome sequencing	CTAB protocol^71^	CTAB

**Critical commercial assays**		

Library prep and sequencing for DNA samples were outsourced to Novogene	https://eu.novogene.com/	150-bp paired-end sequencing NovaSeq X Plus

**Deposited data**		

All trimmed DNA sequence data generated in this study	NCBI Project PRJNA1453165	https://www.ncbi.nlm.nih.gov/bioproject/PRJNA1453165
The analyzed VCF files for the DNA-seq data as well as related scripts	Zenodo	https://zenodo.org/records/19563998

**Software and algorithms**		

R version 4.3.0	R Core Team	https://www.R-project.org/
ggplot2	ggplot2 package^72^	N/A
ggpubr	ggpubr package^73^	N/A
ggsignif	ggsignif package^74^	N/A
Trimmomatic v.0.39	Bolger et al.^75^	N/A
FastQC	FastQC	https://www.bioinformatics.babraham.ac.uk/projects/fastqc/
MultiQC	MultiQC	https://seqera.io/multiqc/
bwa mem for mapping v0.7.17	Li^76^	https://bio-bwa.sourceforge.net/
samtools v1.2.1 for duplicate removal and adding read groups	Li et al.^77^	https://www.htslib.org/
mosdepth v0.3.6 for calculating sequence depth	Pedersen and Quinlan^78^	https://github.com/brentp/mosdepth
bamUtil v1.0.15 function “clipOverlap”	N/A	https://github.com/statgen/bamUtil/releases
freebayes software v1.3.6 to call SNPs	Garrison and Marth^79^	https://github.com/freebayes
script “split_ref_by_bai_datasize.py” to split reference genome	N/A	https://github.com/freebayes/freebayes/blob/master/scripts/split_ref_by_bai_datasize.py
bcftools v1.10 for multiple downstream filtering steps	Danecek et al.^80^	https://samtools.github.io/bcftools/
vcftools v0.1.17 for multiple downstream filtering steps	Danecek et al.^81^	https://vcftools.github.io/
plink v2.00a6.12 for multiple downstream filtering steps	Chang et al.^82^	https://www.cog-genomics.org/plink2/
admixture analysis to estimate individual ancestries	Alexander et al.^83^	https://dalexander.github.io/admixture/
Plot admixture analysis by Joana Meier	N/A	https://github.com/jiangqiuqiuu/speciation_scripts/blob/master/plotADMIXTURE.r
Perl script Fasta2Phylip.pl to create a consensus fasta file	N/A	https://indra.mullins.microbiol.washington.edu/perlscript/docs/Sequence.html
PopArt to create a haplotype network	Leigh and Bryant^84^	https://popart.maths.otago.ac.nz/
Mean comparison of within-mound temperature data using the wilcox-function for Mann-Whitney-U tests	basic stats package	N/A
prop.test - Pearson’s Chi-Squared test for count data to compare mound activity	basic stats package	N/A
linear mixed effect model - lme4 package	Bates et al.^85^	N/A
dHARMa to check model fit	Hartig^86^	N/A
Nagelkerke Pseudo-r^2^ calculation (squaredGLMM function), MuMIn package	Barton^87^	N/A
pairwise taxa comparisons using “emmeans”	Lenth^88^	N/A
Welch’s mean test for mound size	basic stats package	N/A
one-sided Mann-Whitney-U tests for mound size	basic stats package	N/A
Cox regression model (coxph function) - survival package	Thernau^89^	N/A
function “findCorrelation” to keep uncorrelated representative variables (caret package^90^; spearman- method, pairwise-complete.obs)	Kuhn^90^	N/A
Survival model plotted with survfit2 function, ggsurvfit package	Sjoberg^91^	N/A
multinomial logistic regression to test whether taxa have increased using an interaction model; multinom function, nnet package	Venables and BD^92^	N/A
likelihood ratio test “lrtest” to test variable significance; lmtest package	Zeilis and Hothorn^93^	N/A
Non-parametric bootstrap approach, boot package	Canty and Ripley^94^	N/A

**Other**		

Within-mound temperature measurement device	Xavax 111381, Monheim, Germany	N/A
HOBO 64K Pendant Temperature Data Loggers	HOBO	https://www.onsetcomp.com/products/data-loggers/ua-002-64
Eppendorf block heater, Thermomixer R	Eppendorf	022670107
Digital thermometer “Prima”	Amarell	E905052
Climate data from the Finnish Meteorological Institute (FMI)	Aalto et al.^95^	https://paituli.csc.fi/download

### Experimental model and study participant details

#### Animals

All ant samples were collected by the authors of this study. We used female workers for this study because they live longer and conduct all the work in a nest such as taking care of the brood, maintaining the nest, foraging, etc. We collected roughly 60 workers from each mound and selected workers randomly from the nest. During the CT_max_ assays, we used actively-walking ants, which are likely foragers and thus are of a similar age. However, it was not possible to determine the exact age of the workers used, which may have confounded the results. Nevertheless, we argue that by using active workers, these differences are negligible.

This study used ants from a non-threatened and non-protected ant species in Finland. No license or permit was required to conduct these assays. Nevertheless, we still followed the highest ethical standards and international law for ants collected. We also followed two of the 3Rs Principle: (i) We reduced the number of workers collected to a minimum (Reduction principle) and (ii) we created the best accommodation possible for ant workers (Refinement principle). Species or hybrid identity is presented in Table S1.

### Method details

#### Sampling and recording of mound and environmental data

From June to August 2023, *Formica* wood ant mounds were sampled from 92 populations across Finland (Figure 2A, map created using ggplot2,^72^ ggpubr,^73^ and ggsignif^74^ in R v4.3.0 and RStudio v2025.09.0 + 387). From each mound, approx. ten workers were collected alive for thermal tolerance assays (detailed below), and a handful of workers were collected and stored in 96% ethanol for genomic analyses. Whenever possible, workers from the same mound and species were collected in 2023 as used in Satokangas et al.^37^ for genomic and ecological analyses. During sampling, we recorded the within-mound temperature and mound volume for each mound. We measured within-mound temperature from the highest point of the mound using a core thermometer (Xavax 111381, Monheim, Germany; Table S1). We also measured the mound length, width, and height using a measuring tape and calculated the mound volume following^96^ and using the equation: 2/3 × π × D/2 × d/2 × H where D is the length, d is the width, and H the height of the mound.

#### Genomic analyses

##### DNA extraction, whole-genome sequencing, and variant identification

To identify hybrids and different species, we extracted DNA from a single diploid worker per mound and population (*N* = 98; after filtering *N* = 92) using the Qiagen DNeasy Blood and Tissue kit (Qiagen, Hilden, Germany; *N* = 81) following the manufacturer’s protocol or a modified CTAB extraction protocol (*N* = 17;^71^). In both procedures, DNA was eluted in 100 μL AE elution buffer from the Qiagen kit. Library preparation and whole-genome sequencing were outsourced to a commercial provider (Novogene, UK). Whole-genome sequencing was conducted on an Illumina NovaSeq X Plus with 150bp paired-end sequencing.

Raw Illumina reads and adapter sequences were trimmed using trimmomatic (v0.39;^75^), sequence quality was checked using FastQC (https://www.bioinformatics.babraham.ac.uk/projects/fastqc/) and MultiQC (https://seqera.io/multiqc/). We then mapped sequences mapped against a Finnish *F. aquilonia* × *F. polyctena* hybrid reference genome^97^ using bwa mem (v0.7.17;^76^). Using picard tools (v2.27.5; http://broadinstitute.github.io/picard), duplicates were removed with the command “MarkDuplicates”, read groups were added with the command “AddOrReplaceReadGroups”, and bam files were indexed using “samtools index” in samtools v1.2.1.^77^ The average read depth was 15.7×, as computed across individuals using mosdepth (v0.3.6;^78^) after duplicate removal. Overlaps were clipped using “bamUtil” and the function “bam clipOverlap” (v1.0.15, https://github.com/statgen/bamUtil/releases).

##### SNP calling and filtering data

For single nucleotide polymorphism (SNPs) calling, we used 98 samples from the ‘current’ sampling plus additional 85 samples from a so-called ‘previous’ sampling from Satokangas et al. (2023)^37^ as a reference. We called SNPs with the freebayes software v1.3.6.^79^ To speed up the SNP calling, 50 kb regions of the reference genome were defined using the script “split_ref_by_bai_datasize.py” (available under https://github.com/freebayes/freebayes/blob/master/scripts/split_ref_by_bai_datasize.py; main settings: --k option to disable population priors; --genotype-qualities to write genotype qualities to a file; --skip-coverage to skip high coverage regions; --limit-coverage 100 to only use regions with a maximum coverage of 100; --use-best-*n*-alleles 3 to only consider 3 alleles to reduce runtime). The resulting VCF file was normalized using bcftools norm-function (v1.10;^80^). Using bcftools, sites located at less than two base pairs from InDels were excluded, as well as sites that were supported by only Forward or Reverse reads. Furthermore, we only kept biallelic SNPs with quality values equal to or higher than 30 and, we filtered sites based on individual sequencing depth: Genotypes were marked as missing when their depth exceeded twice the mean depth of the individual in question. Genotyping errors (e.g., misaligned reads) were removed by excluding sites that displayed heterozygote excess after pooling all samples using vcftools v0.1.17.^81^ Individual genotypes with a quality of lower than or equal to 30 and a depth of coverage lower than or equal 8 were set as missing data. Sites with more than 10% of missing data over all samples were removed from the next steps. Lastly, singletons were removed (minor allele count <2) using vcftools.

After these filtering steps, 3,324,311 SNPs were kept from 179 individuals with sequencing depths of at least 8×, including 92 out of the 98 samples from the ‘current’ sampling in 2023. Using vcftools, the final dataset was thinned with 20 kb distances to minimize linkage disequilibrium. We further excluded chromosome three (the social chromosome with reduced recombination due to supergene inversions;^98^) as well as chromosome ‘zero’, which contains all unscaffolded contigs with no known genomic location.^97^ The final dataset resulted in 9,979 genome-wide SNPs used for the genomic analyses described below.

#### Species and hybrid identification

##### Principal component analysis (PCA) and admixture analyses

To assess the species’ identity, genomic structure and hybrid status, a principal component analysis (PCA) was conducted in plink (v2.00a6.12,^82^) using the nuclear genomic SNPs data from all samples. We expected that genomically diverged species and hybrids would form separate clusters in the PCA (Figure S1A).

To assess the extent of admixture and thus hybridization in the samples, we performed an admixture analysis^83^ with the 9,979 nuclear SNP data. For admixture, the number of clusters K was chosen to range from K = 2 to 10, without prior knowledge of the taxa but including samples and species identity from the ‘previous’ sampling.^37^ The most likely value of K was chosen based on the lowest cross-validation error rates. We further extracted the allele frequencies from the most likely K to be used in statistical analyses (Figure S1B). The admixture analysis was displayed by custom-modifying the “plotAdmixture.R” script by Joana Meier (https://github.com/jiangqiuqiuu/speciation_scripts/blob/master/plotADMIXTURE.r).

##### Mitochondrial haplotype network

To create a haplotype network using the mitochondrial DNA only, we called SNPs from the mitochondrial genome using all 179 samples. Specifically, we used slightly different settings than with the nuclear DNA, namely --skip-coverage 15200 --limit-coverage 100 --use-best-*n*-alleles 3 --pooled-continuous. We used the same filtering steps as detailed above with the nuclear DNA. After filtering, 466 SNPs were kept. A consensus sequence for each sample across all samples was created by converting the VCF file into a fasta-file using the “faidx” function in samtools and the VCFtools script “vcf-consensus”. Next, the fasta-file was converted to a phylip-format using the perl script “Fasta2Phylip.pl” (https://indra.mullins.microbiol.washington.edu/perlscript/docs/Sequence.html) and used to create a median-joining network in PopArt.^84^ Species that share identical or similar mitochondrial haplotypes should cluster more closely together in the network (Figure S2).

##### Selecting hybrids and *F. aquilonia* for subsequent comparisons

After reliably identifying species and hybrids, we only used samples from *F. aquilonia* × *F. polyctena* hybrids and its cold-adapted *F. aquilonia* parent for subsequent analyses. We did not find the warm-adapted parent *F. polyctena* in the dataset. We also excluded the warm-adapted *F. rufa* and *F. pratensis*, as well as cold-adapted *F. lugubris* because they are either single-queened or have facultatively have only a few queens and are not as dominant locally as *F. aquilonia* or *F. aquilonia x F. polyctena* hybrids. Both *F. aquilonia* and hybrids are also socially more similar (i.e., both are multiple-queened and form supercolonies,^99^).

#### Downstream genomic analyses

##### Calculating observed heterozygosity, nucleotide diversity, hybrid index, and intraclass heterozygosity

To assess whether hybrids had a greater genomic diversity than *F. aquilonia*, we first calculated the observed heterozygosity for each sample from the current sampling using vcftools --het and then tested if their means differed significantly using a Welch’s *t* test. To calculate the genome-wide nucleotide diversity for *F. aquilonia* and hybrids separately, we calculated values using vcftools --site-pi and then averaged values over the genome. We compared the data using a Welch’s *t* test. To calculate the hybrid index for the hybrid samples, we used the ‘previous’ and ‘current’ sampling and filtered the VCF-file to only keep the parental species *F. aquilonia* and *F. polyctena* and the *F. aquilonia* × *F. polyctena* hybrids. Then, we removed monomorphic sites, selected ancestry informative markers (AIMs) with an allele frequency difference of 0.95 between *F. aquilonia* and *F. polyctena*,^97^ and filtered the data to keep only markers with less than 5% missing data. Lastly, we thinned the VCF file to 1 SNP per 500 kb yielding 359 SNPs. We then exported the VCF file and calculated the hybrid index for each individual as following: sum of *F. aquilonia* alleles/((sum of homozygous *F. aquilonia* and homozygous *F. polyctena* alleles) - 2 ∗ number of missing genotypes; Figure S3A). Using the hybrid index and individuals from the ‘current’ sampling 2023, we conducted a linear regression testing whether the hybrid index is associated with the latitude as well as a linear regression testing whether it is associated with the temperature when ants fall into heat coma (section “*Critical Thermal Maximum (CT*_*max*_*) assays*”; Figures S4E and S7E). We also calculated the intraclass heterozygosity for each individual as following: sum of heterozygous alleles/(sum of homozygous *F. aquilonia* alleles, sum of heterozygous alleles, and sum of homozygous *F. polyctena* alleles).

##### Identifying mtDNA origin and pairwise mtDNA mutations

Using the mtDNA haplotype network, we identified the maternal species from which hybrids originated (i.e., *F. aquilonia* or *F. polyctena*) as mtDNA is maternally inherited and calculated pairwise mtDNA mutations for each sample pairs. Using an Analysis of Variance (ANOVA), we tested whether the mtDNA origin is associated with the latitude (Figure S7F). Further, we correlated the pairwise mtDNA mutations for the hybrid maternal lineages against the geographic distance using a linear regression to test whether mtDNA mutations are correlated with latitude (Figures S3B and S7B).

#### Statistical analyses of long-term and cross-sectional environmental data

##### Long-term monitoring and analyses of mound activity, number of reproductive offspring, and environmental variables between 2020–2023

Between 2020 and 2023, the activity of wood ant mounds, the emergence time and number of reproductive offspring (i.e., new queens and males), as well as the within-mound temperature were recorded from March to June in six populations Southern Finland. We observed three *F. aquilonia* populations (*Haltiala*, *Pusula*, *Soleböle*) and three *F. aquilonia* × *F. polyctena* hybrid populations (*Bunkkerikal*, *Langholmen*, *Svanvik*).

The within-mound temperature was measured using HOBO data loggers (HOBO 64K Pendant Temperature Data Logger, Bourne, USA). To analyze the within-mound temperature, we first calculated means for each month over the three observation years for hybrids and *F. aquilonia* separately. As the data was not normally distributed, we compared the means using Mann-Whitney-U tests.

The mound activity was recorded as a binomial (1 = active; 0 = non-active). We further recorded the date and counted and/or estimated the number of sexual offspring present on the mound (counted with 1–20 individuals; estimated with >20). For both mound activity and number of reproductive offspring, we calculated the relative percentages (i.e., relative proportions calculated over all years; count data for focal species/count data for both species). We used a proportion test (prop.test) for the mound activity to test whether the mound activity differed between hybrids and *F. aquilonia* for each month separately. We used a Pearson’s Chi-Squared test for count data to compare the number of estimated reproductives under the null hypothesis of no difference between the species for each month separately.

##### Analyses of cross-sectional environmental variables recorded during sampling

During sampling, we recorded the temperature within-mounds and mound volume (detailed above). To assess whether hybrids and *F. aquilonia* differ in these variables in the sympatric area (i.e., where both *F. aquilonia* and hybrids co-occur; latitude lower than 63.99), we compared the variables separately in a linear mixed effect model (LMER, lme4 package^85^; Figures S7A, S7B, and S7D). The explanatory variables were the genomic assignment of taxa and the latitude of each sample, and the random factors were the population ID and the date of sampling. The model fit was checked using dHARMa.^86^ To calculate r^2^ values for each model, we created an intercept-only model for each variable and included the same random effects. Specifically, we calculated the Nagelkerke Pseudo-r^2^ (squaredGLMM function, MuMIn package^87^). Such a Pseudo-r^2^ value is a relative measure among similar models indicating how well the model explains the data and shows values for the fixed effects as well as fixed and random effects together. To check whether the species differed in the variables, we calculated pairwise contrasts (emmeans, adjusting for multiple testing;^88^). We further tested whether the mound volume is associated with the latitude by correlating both in a linear regression – separately for hybrids and *F. aquilonia*. For this, we excluded one hybrid and one *F. aquilonia* outlier mounds with extremely large dimensions based on *Z* score outlier tests conducted for both taxa separately. We then split the hybrid and *F. aquilonia* datasets into a “southern” and “northern” area based on their respective latitudinal means and compared the means within taxa using a Welch’s *t* test for hybrids and a Mann-Whitney-U-test for *F. aquilonia* (data not normally distributed). Using one-sided Mann-Whitney-U tests, we also tested whether hybrid and *F. aquilonia* mounds differ overall in the sympatric range and across entire Finland.

#### Critical thermal maximum (CT_max_) assay and statistical analyses

##### Conducting the CT_max_ assay

From each mound, four workers were used in an CT_max_ assay using a dry bath (Eppendorf block heater, Thermomixer R, Hamburg, Germany). We chose a dry bath because body size does not influence heat tolerance in a dry bath assay compared to using a hot plate.^56^^,^^100^ To avoid potential experimental acclimation, we tested the ants on the same day as they were sampled.^101^ Workers were transferred individually to an Eppendorf tube and sealed with cotton briefly before the start of the experiment to minimize the time each worker spent inside the tube. The tubes with workers were then placed in a block heater, which marked the start of the experiment. Workers were exposed to 40 °C for 4 min (Figure S4A inset). The temperature was then gradually increased to 42, 45, 47, and finally 49 °C. These temperature steps were held for approximately 3 min. Between temperature steps, the temperature of the block heater increased to the respective temperature in 1 or 2 minutes. The temperature steps were selected based on pilot studies. In total, the assay lasted 20 min. As a control, we transferred one worker from each population individually to a single tube, sealed it with cotton, and kept it at room temperature to ensure that workers do not fall into a coma and die due to other circumstances.

Each minute, the state of the workers inside the tubes was recorded, with workers either being “active” or “knocked down”. This procedure was repeated until four workers from each mound were tested. A digital thermometer (“Prima”, Amarell, Kreuzwertheim, Germany) was placed inside a tube and sealed with cotton to measure the temperature inside the tube during the assay. This temperature was recorded at the beginning of each minute. The order in which the workers were placed in the dry bath was block-randomized to minimize potential heating differences of the block heater. To reduce any batch effects, at least one worker from each colony was tested in the same CT_max_ run.

##### Survival analysis and statistical associations with mtDNA and hybrid index

To check if ants from hybrid and *F. aquilonia* populations differ in the CT_max_ assay, we calculated proportion hazards of ants falling into heat coma using a Cox regression model (coxph function; survival package^89^). Such a Cox regression model allows using multiple fixed factors and a random factor. We used the species/hybrid identity, the parental mtDNA status (*F. polyctena* or *F. aquilonia* for hybrids; only *F. aquilonia* for *F. aquilonia*), the hybrid index, as well as weather data prior to sampling (see the next paragraph for details on the weather data). We scaled all continuous data (the hybrid index and weather data) to make them comparable and used the latitude (of where mounds where collected) as random factor using the “frailty” function (survival package).

We calculated weather data prior to sampling for each mound by extracting temperature and precipitation data 21 days prior to sampling until the day of sampling from the Finnish Meteorological Institute data. We downloaded temperature and precipitation data from the Finnish Meteorological Institute (FMI;^95^) and extracted the data for each mound from digital rasters (FMI ClimGrid gridded daily climatology) at a 10-km spatial resolution for up to six years before the sampling year. We then calculated the mean, minimum, maximum, and range (max–min) temperature and precipitation of 21 days including the day of sampling, 14 days + day of sampling, 7 days + day of sampling, 5 days + day of sampling, 3 days + day of sampling, one day before sampling, and the day of sampling. As some variables are correlated, we excluded highly correlated values using the function “findCorrelation”, which keeps the most uncorrelated representative variables (caret package; spearman-method, pairwise-complete.obs). From these variables, we kept the maximum and minimum temperature 21 days prior to sampling including the day of sampling, the minimum temperature 5 days prior to sampling, temperature range 7 and 3 days prior to sampling, maximum precipitation values 1 day prior to sampling, and the precipitation range 7 days prior to sampling.

Further, we conducted a non-parametric Kaplan-Meier survival analysis. For this, the first timepoint when a worker was recorded as knocked-down was selected and analyzed (surv function, survival package,^89^; plotted with survfit2 function, ggsurvfit package^91^; Figures S4A and S4B). We conducted the survival analysis with the state of the worker as a response variable, and the time and the species/hybrid identity as explanatory variables (time and temperature were highly correlated; r^2^ = 0.93, t = 41.25, df = 281, *p*-value <0.001). We then extracted the survival probabilities for minutes 8–12 from the survival analysis. These minutes encompass temperature values around 45 °C, which represent a biologically relevant temperature, when *Formica* ants fall into heat coma (identified in a pilot study, data not shown). To account for climatic changes but still compare hybrid and *F. aquilonia*, we conducted two approaches: In the first approach, we only selected samples from a sympatric area (i.e., latitude lower than 63.9), and in the second approach, samples from entire Finland.

As we found hybrids that were sired from either parent, *F. aquilonia* or *F. polyctena*, we tested whether the temperature at which ants fall into heat coma differs between the maternal lineages using a one-sided Mann-Whitney-U test (Figures S4C and S4D). Additionally, we tested whether the hybrid index is correlated with the temperature at which ants fall into heat coma using a linear regression (Figure S4E).

#### Persistence of hybrids and their expansion identified by 5-year climatic averages

##### Number and persistence of hybrids and geographic extent of hybrids

In the current sampling 2023, we re-visited 42 out of 75 populations from the ‘previous sampling'^37^ that were already known to assess the number and persistence of hybrids as well as the geographic extent of hybridization. Whenever possible, workers from the same mound were sampled, or workers from mounds within a distance of 500 m, when the focal known mound was abandoned. Based on the taxa identity (i.e., species or hybrid) retrieved in both studies, we checked if the taxa persisted or changed. We also calculated the number of *F. aquilonia* × *F. polyctena* hybrid samples in both datasets and checked the latitude between datasets where hybrid populations were found. To check whether the frequency of hybrid samples has increased over time and with latitude conducted a multinomial logistic regression (Figure S4F). For this, we lumped all cold-adapted (*F. aquilonia, F. lugubris*) and all warm-adapted (*F. pratensis, F. rufa*) species together and used the taxa identity (i.e., hybrids as well as cold- and warm-adapted species) as the response variables and the time (as factor) and latitude (as continuous variable) as explanatory variables (interaction model; multinom function, nnet package^92^). We then checked whether variables contributed significantly to the model by conducting a Likelihood ratio tests (lmtest package^93^). Lastly, we manually calculated a *Z* score (i.e., Wald Z), two-tailed z-test, and a Nagelkerke Pseudo-r^2^ value for the model values. To check the model fit, we compared the model’s Akaike Information Criterion for small sample sizes (AICc) with the AIC of an intercept-only model.

##### Calculation and statistical analyses of the 5-year climatic averages

To calculate climatic changes over time for the sampling locations, we used the downloaded temperature and precipitation data from the Finnish Meteorological Institute (FMI). Again, we extracted the data for each mound from digital rasters (FMI ClimGrid gridded daily climatology) at a 10-km spatial resolution for up to six years before the sampling year. We then calculated averages over 5 years before the respective sampling year. For example, if a mound from population was sampled in 2023, the climate for this mound was averaged over the years from 2018 to 2022. If it was sampled in 2005, it was averaged over the years from 1999 to 2004, etc. We calculated six climatic variables, (i) winter mean temperature, (ii) spring mean temperature, (iii) winter mean precipitation, (iv) spring mean temperature, (v) the number of days above 0 °C in winter, and (vi) the number of days below 0 °C in spring (Figures S6A–S6F). Winter and spring were defined as the months of December to February and the months of April and May, respectively, and identically calculated as in Satokangas et al.^37^ for comparability.

For the climatic change analysis, we used the ‘current' sampling as well as only samples from 2005 originating from the ‘previous' sampling. We then defined three ranges. A hybrid range where hybrids were found in 2005 (i.e. ‘established' range), an ‘expanded' range at higher latitudes where we found hybrids in 2023, and an ‘expanded' range at higher latitudes where we did not find hybrids in 2005. Our rationale was as follows: If climatic changes have allowed a northward expansion of hybrids, we expect that the climate in the expanded range has changed in a way that is favourable for hybrids. Specifically, we expect that the expanded range in 2023, where hybrids have been found, should have a similar climate compared to that of the hybrid range in 2005.

We then conducted three comparisons. (i) To test whether the climate has changed and allowed a northward expansion of hybrids, we compared the climate of hybrid populations in the ‘established' hybrid range found in 2005 (lower latitudes, range = 59.92 to 62.56, Figure 2B, light purple color; *N* = 5;^37^) with the climate of hybrid populations in the ‘expanded’ range in 2023 at higher latitudes (latitudinal range = 62.57 to 63.97, Figures 2B and S5A–S5D, dark purple color; *N* = 4). (ii) We also compared the climate of hybrid populations in the ‘established' hybrid’ range found in 2005 (lower latitudes, range = 59.92 to 62.56, Figure 2B, light purple color) with the climate of hybrid populations in the ‘expanded’ range but using climatic data from 2005 where hybrids were absent (latitudinal range = 62.57 to 63.97, Figure 2B, yellow color; *N* = 4). (iii) Lastly, we compared the climate of hybrid populations in the ‘expanded’ range in 2023 (higher latitudes, range = 62.57 to 63.97, Figure 2B, dark purple color) with the climate of hybrid populations with the climate of hybrid populations in the ‘expanded’ range but using climatic data from 2005 (latitudinal range = 62.57 to 63.97, Figure 2B, yellow color). Hybrids with different mtDNA parental species were combined in the comparison because they did not differ in the environmental variables.

Due to a low sample size in each range (N = 4–5), we conducted a non-parametric bootstrap approach (library ‘boot’^94^). For each comparison, we re-sampled the data 1,000 times with replacement and calculated the difference in the means. To calculate a *p*-value, we extracted the 95% confidence intervals from the mean differences and counted the bootstrap values in which the mean difference was on the opposite side of zero from the observed mean, which indicates a significant difference between the data. We corrected the results for multiple testing by applying a Holm-Bonferroni correction.

We further tested whether the climate has overall changed in both hybrid and *F. aquilonia* populations over 18 years. For this, we compared the same six environmental variables described above within and between hybrid and *F. aquilonia* populations and between previous and current sampling. We compared the environmental variables using t-tests (if normally distributed) or Mann-Whitney-U tests (if not-normally distributed) and corrected the results for multiple testing.

## Supplemental information

Document S1. Figures S1–S7 and Tables S2–S8

Table S1. Mound GPS coordinates and characteristics recorded during fieldwork

Data S1. Data1.xlsx. Excel file with all relevant data to reanalyze the main results

Data S2. Wood_ants_Finland_MS.R. R-script to reanalyze the main results

## Footnotes

Supplemental information can be found online at https://doi.org/10.1016/j.isci.2026.117834.
