# Supplementary material for: Evidence for climate-mediated range expansion of hybrid wood ants
Source: iScience. 2026 Oct 15;29(11):117834. doi: 10.1016/j.isci.2026.117834 (PMC13691259; doi:10.1016/j.isci.2026.117834)
Supplement: Supplementary file 1 [file mmc1.pdf]

iScience, Volume *29*

Supplemental information

Evidence for climate-mediated range expansion of hybrid wood ants

Patrick Krapf, Patrick Heidbreder, Marjolein Bruijning, Patrick G. Meirmans, Sean Stankowski, and Jonna Kulmuni

This PDF file includes:

Figs. S1 to S7
Tables S2 to S8
Data S1 to S2

Other Supplementary Materials for this manuscript include the following:

Data S1 to S2

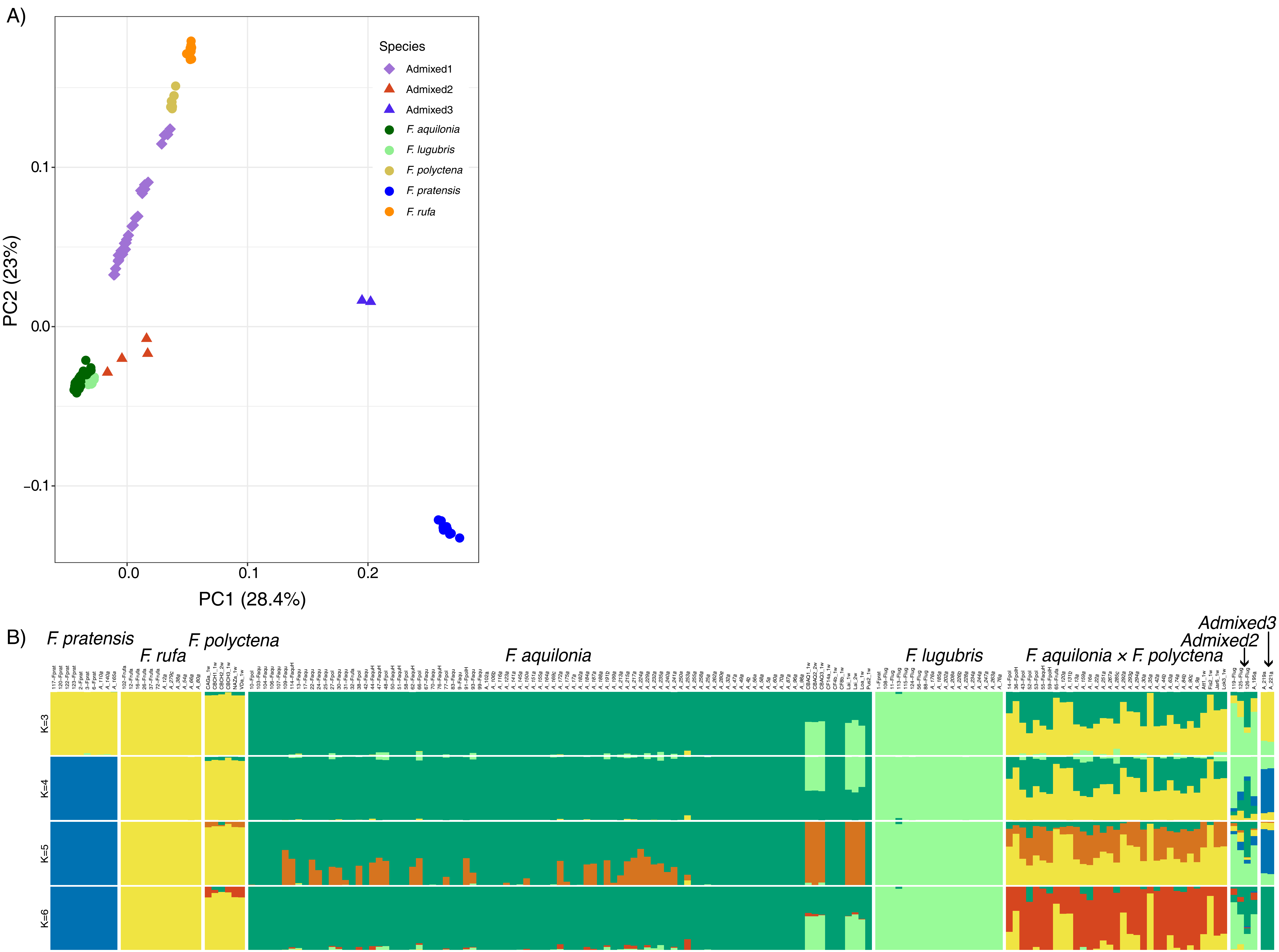
A)
Species
Admixed1
Admixed2
Admixed3
F. aquilonia
F. lugubris
F. polyctena
F. pratensis
F. rufa
PC2 (23%)
PC1 (28.4%)
0.1
0.0
−0.1
0.0
0.1
0.2
B)
F. pratensis
F. rufa
F. polyctena
F. aquilonia
F. lugubris
F. aquilonia × F. polyctena
Admixed2
Admixed3
K=3
K=4
K=5
K=6

Fig. S1. Downstream analyses of the whole-genome data for the five wood ant species and hybrids. (A) PCA with species assignment. In the PCA, *F. pratensis* samples clustered separately from other species and hybrid samples on the first axis (28.4%); only Admixed3 samples clustered nearby and were also separated from the other samples. The second axis (23%) separated mainly cold-adapted species *F. lugubris and F. aquilonia* from warm-adapted species *F. polyctena* and *F. rufa* and hybrid samples. Cold-adapted species *F. lugubris* and *F. aquilonia* clustered closely together, and Admixed2 samples also clustered nearby to them, although more loosely aggregated. Warm-adapted species *F. polyctena* and *F. rufa* also clustered closely together but separately for each species, while Admixed1 (*F. aquilonia* × *F. polyctena* hybrids) samples clustered loosely between *F. aquilonia* and *F. lugubris* as well as *F. polyctena* and *F. rufa*. (B) In the admixture plot, samples were identified as mainly 'pure' species and admixed taxa, and the same assignment of samples was retrieved as in the PCA. Each column represents a sample and are organised by species or hybrid identity. Related to Figure 1.

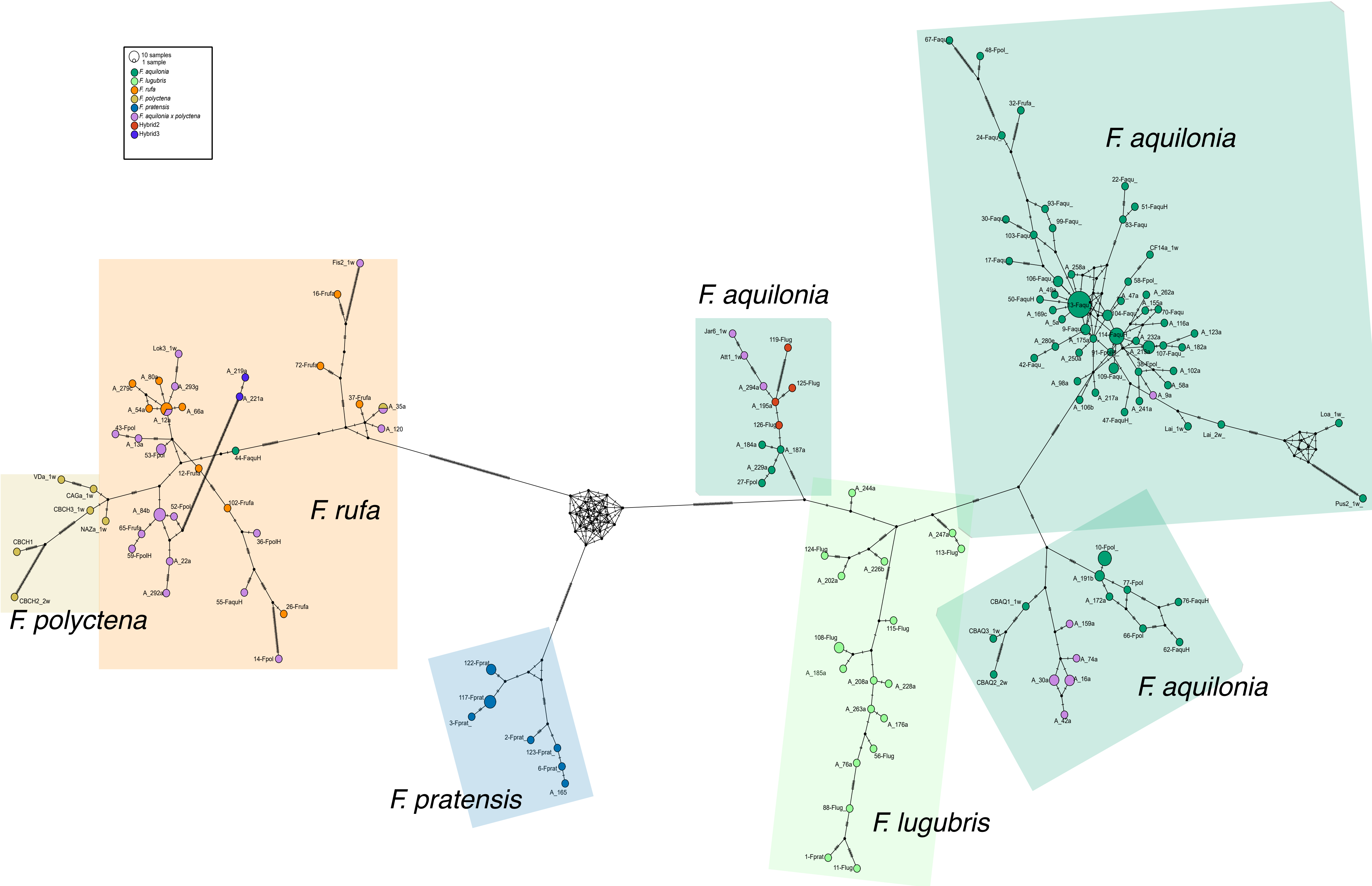

10 samples
1 sample
F. aquilonia
F. lugubris
F. rufa
F. polyctena
F. pratensis
F. aquilonia x polyctena
Hybrid2
Hybrid3
F. aquilonia
F. aquilonia
F. rufa
F. polyctena
F. pratensis
F. lugubris
F. aquilonia

Fig. S2. Mitochondrial haplotype network. A minimum spanning network was created in PopArt using all 2023 and reference samples[37] ($N_{indsTotal}$=179). For this, we called variants specifically on the mitochondrial DNA (N_{SNPs}=466). The dots represent individual haplotypes, dot sizes represent the frequency of specific haplotypes, dot colours represent the nuclear genomic ancestry (N_{SNPs}=9979), while coloured areas around the dots represent species clustering. Dashes represent the number of mutations between the haplotypes. Individual IDs are given as well. The mitochondrial haplotypes cluster mainly by species, although *F. polyctena* and *F. rufa* cluster closely together. Related to Figure 1.

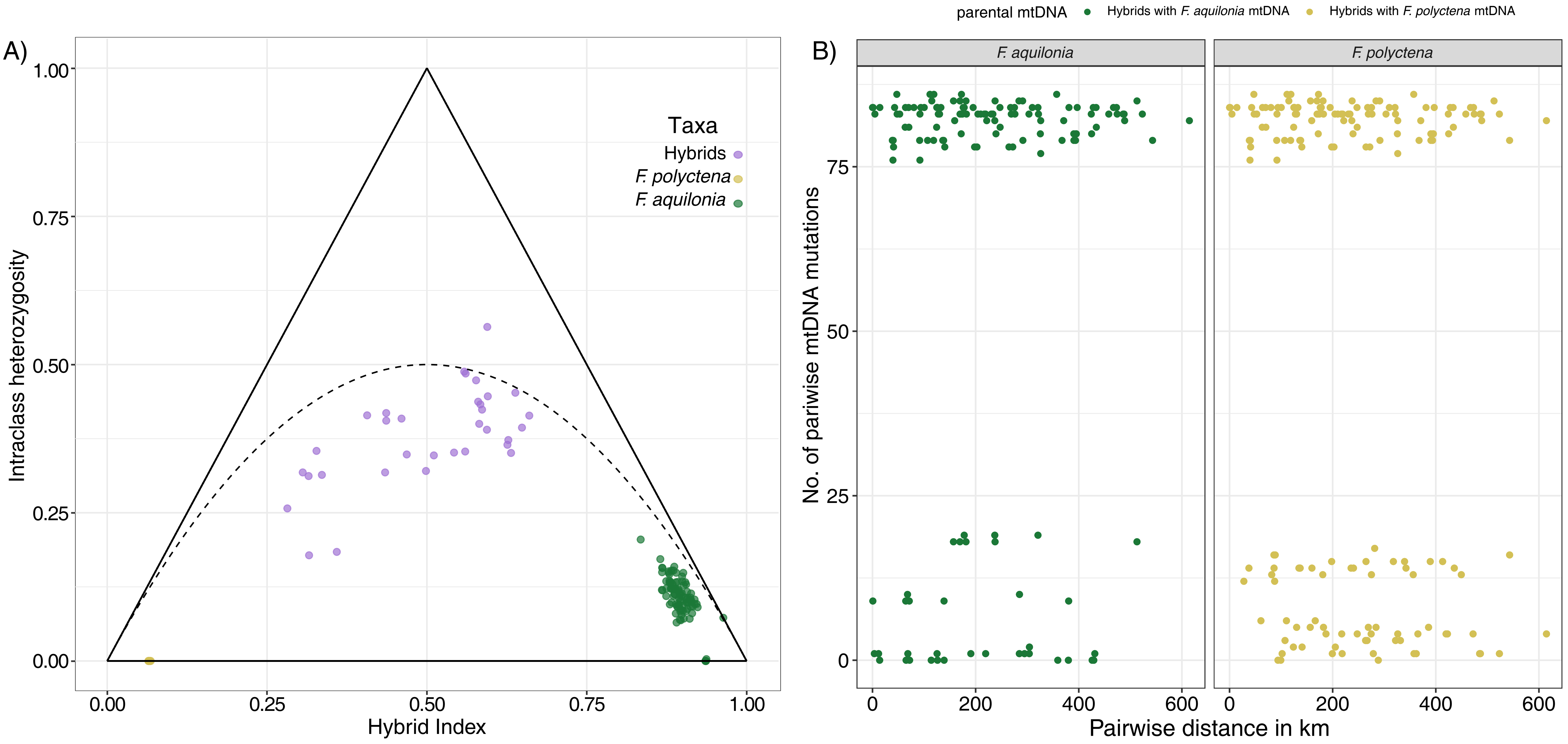

A)
Taxa
Hybrids
F. polyctena
F. aquilonia
Intraclass heterozygosity
Hybrid Index
0.00
0.25
0.50
0.75
1.00
B)
parental mtDNA
Hybrids with F. aquilonia mtDNA
Hybrids with F. polyctena mtDNA
F. aquilonia
F. polyctena
No. of pairwise mtDNA mutations
Pairwise distance in km
0
25
50
75
200
400
600

Fig. S3. The (A) hybrid index and (B) number of mutations between hybrid sample pairs. Related to Figure 1.

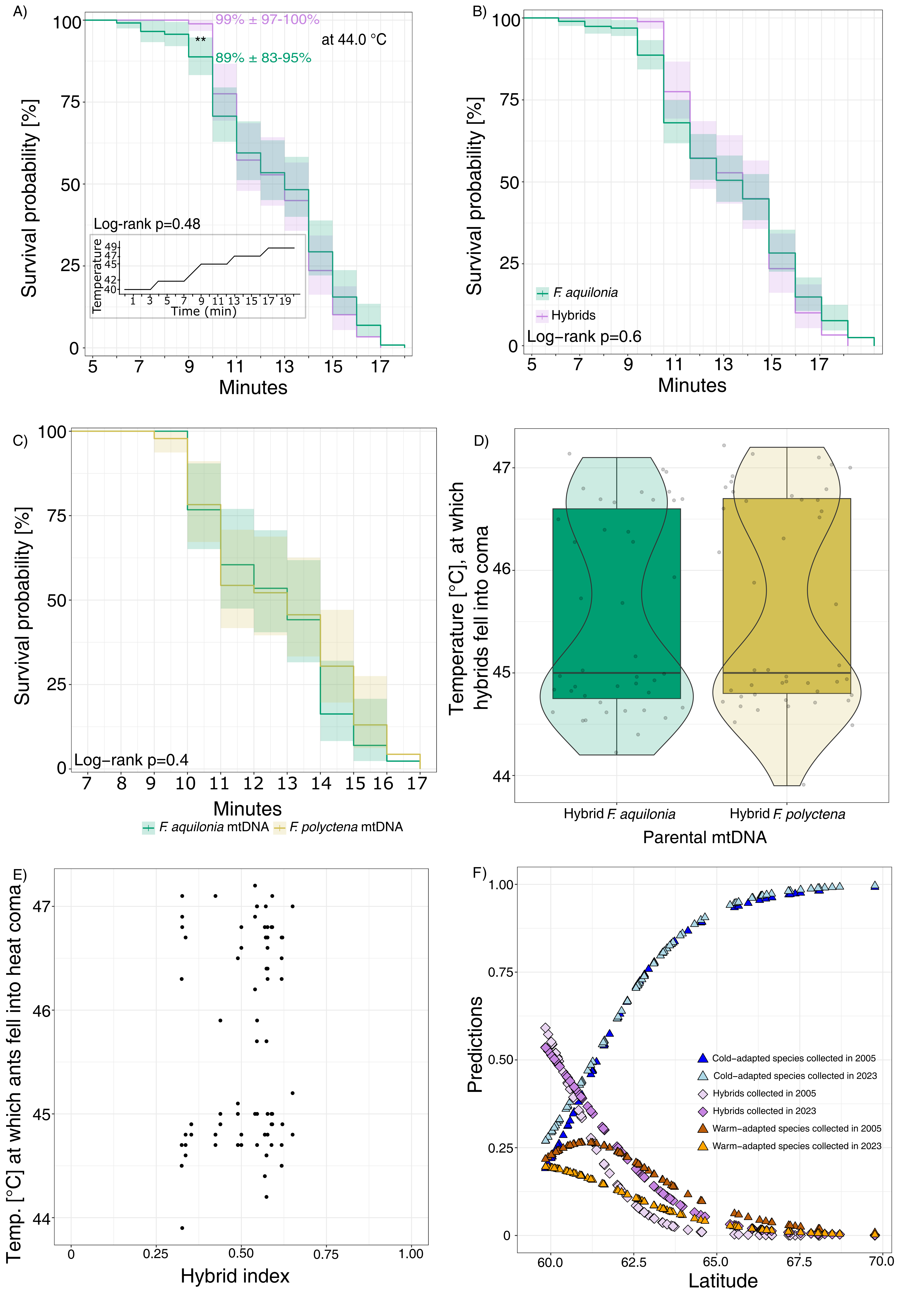

A)
99% ± 97-100%
89% ± 83-95%
**
at 44.0 °C
Survival probability [%]
Log−rank p=0.48
Temperature
Time (min)
Minutes
B)
Survival probability [%]
F. aquilonia
Hybrids
Log−rank p=0.6
Minutes
C)
Survival probability [%]
Log−rank p=0.4
Minutes
F. aquilonia mtDNA
F. polyctena mtDNA
D)
Temperature [°C], at which hybrids fell into coma
Hybrid F. aquilonia
Hybrid F. polyctena
Parental mtDNA
E)
Temp. [°C] at which ants fell into heat coma
Hybrid index
F)
Predictions
Cold−adapted species collected in 2005
Cold−adapted species collected in 2023
Hybrids collected in 2005
Hybrids collected in 2023
Warm−adapted species collected in 2005
Warm−adapted species collected in 2023
Latitude

Fig. S4. CT_{max} assay results between *F. aquilonia* and hybrids and detailed analysis of hybrids as well as the plot of the multinomial logistic regression. (A) Survival probabilities from the survival analysis for hybrids and *F. aquilonia* in the sympatric area (workers tested, N hybrids = 89; N *F. aquilonia* = 116). The asterisks represent differences between the survival probabilities at minute 9 (43.9 °C; test of given proportions; p-value ** <0.01) with respective survival probabilities for hybrids and *F. aquilonia*. The inset in (A) displays the CT_{max} temperature increase over 20 minutes. (B) No significant difference in the survival analysis between hybrids at *F. aquilonia* in entire Finland (Kaplan-Meier survival analysis). (C) No significant difference in the survival analysis between hybrids that originated from either *F. aquilonia* or *F. polyctena* maternal lineages (maternal mtDNA; Kaplan-Meier survival analysis). (D) Hybrids with *F. polyctena* mtDNA fall into heat coma at a similar temperature as hybrids with *F. aquilonia* mtDNA. The interquartile range (25th-75th percentiles) is represented by the box, while the median is represented by the line within the box. Most extreme observations within the 1.5 times the interquartile range below and above the percentiles are presented with whiskers. Observations beyond the whiskers are presented as dots. (E) No correlation between the hybrid index and the temperature, at which ants fall into heat coma. (F) Predictions of the multinomial logistic regression to find warm- and cold-adapted species and hybrid populations at higher latitudes when comparing the 'previous' sampling conducted in 2005 with the 'current' sampling conducted in 2023 conducted using a multinomial logistic regression. Related to Figure 1.

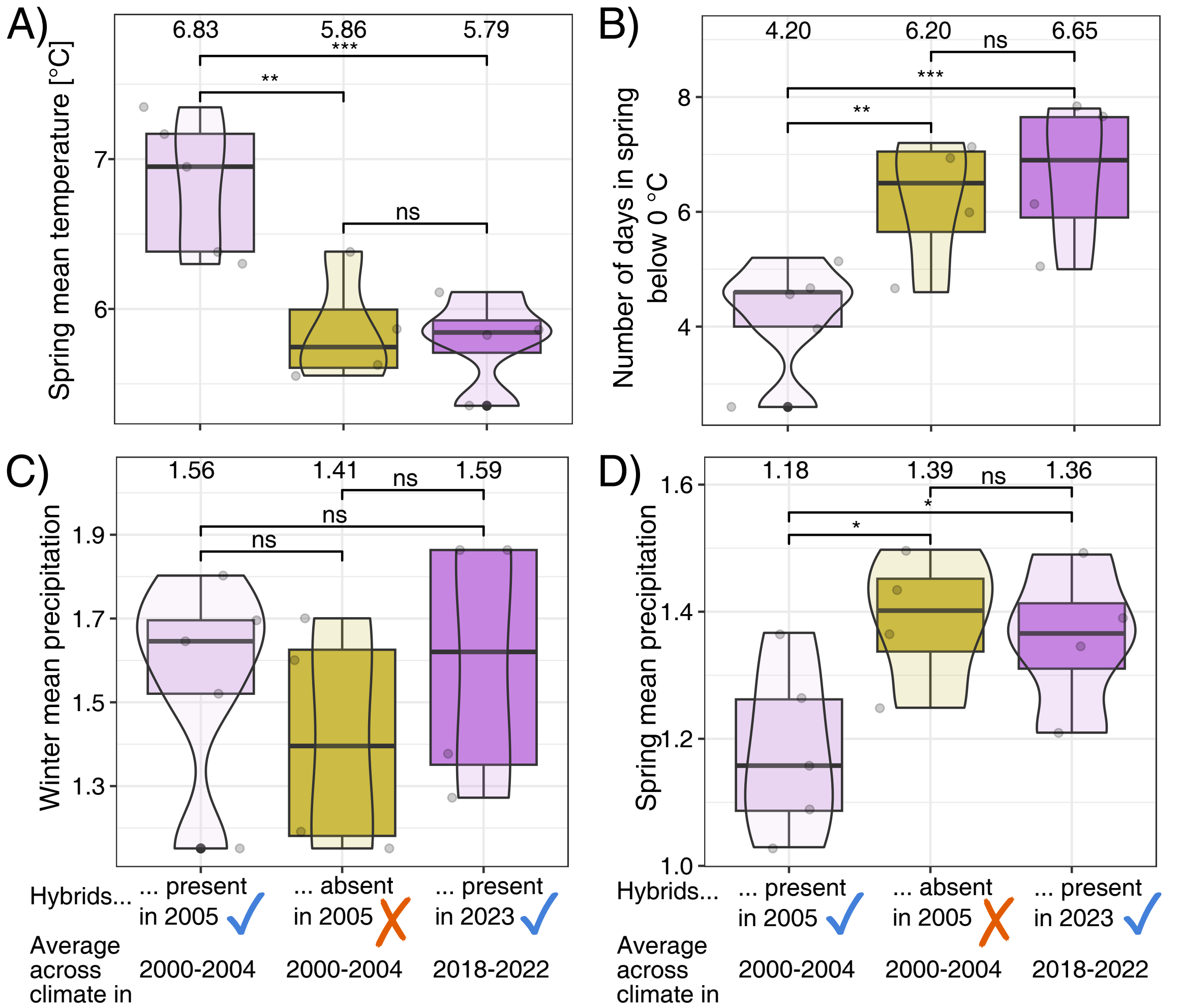

A)
6.83
5.86
5.79
***
**
ns
Spring mean temperature [°C]
7
6
B)
4.20
6.20
6.65
ns
***
**
Number of days in spring below 0 °C
8
6
4
C)
1.56
1.41
1.59
ns
ns
ns
Winter mean precipitation
1.9
1.7
1.5
1.3
D)
1.18
1.39
1.36
ns
*
*
Spring mean precipitation
1.6
1.4
1.2
1.0
Hybrids...
... present in 2005
... absent in 2005
... present in 2023
Average across climate in
2000-2004
2000-2004
2018-2022

Fig. S5. Climatic 5-year averages of four environmental variables are plotted for the hybrid populations only. The variables were calculated backward from the sampling year 2005 (average across climate 1999-2004) and 2023 (average across climate 2018-2022). (A) Spring mean temperature. (B) Number of days in spring below 0 °C (frosty spring days). (C) Winter mean precipitation. (D) Spring mean precipitation. 'Winter' is defined as the diapause period between December to February and 'Spring' as the reproductive period between April to May. Values above the boxplots represent mean values. Asterisks represent bootstrapped p-values between hybrid areas at higher and lower latitudes and over time (Tab. S5) with * <0.05, ** <0.01, and ***<0.001. N suitable in 2005 = 5. N unsuitable in 2005 = 4. N suitable in 2023 = 4. The interquartile range (25th-75th percentiles) is represented by the box, while the median is represented by the line within the box. Most extreme observations within the 1.5 times the interquartile range below and above the percentiles are presented with whiskers. Observations beyond the whiskers are presented as dots. Related to Figure 2.

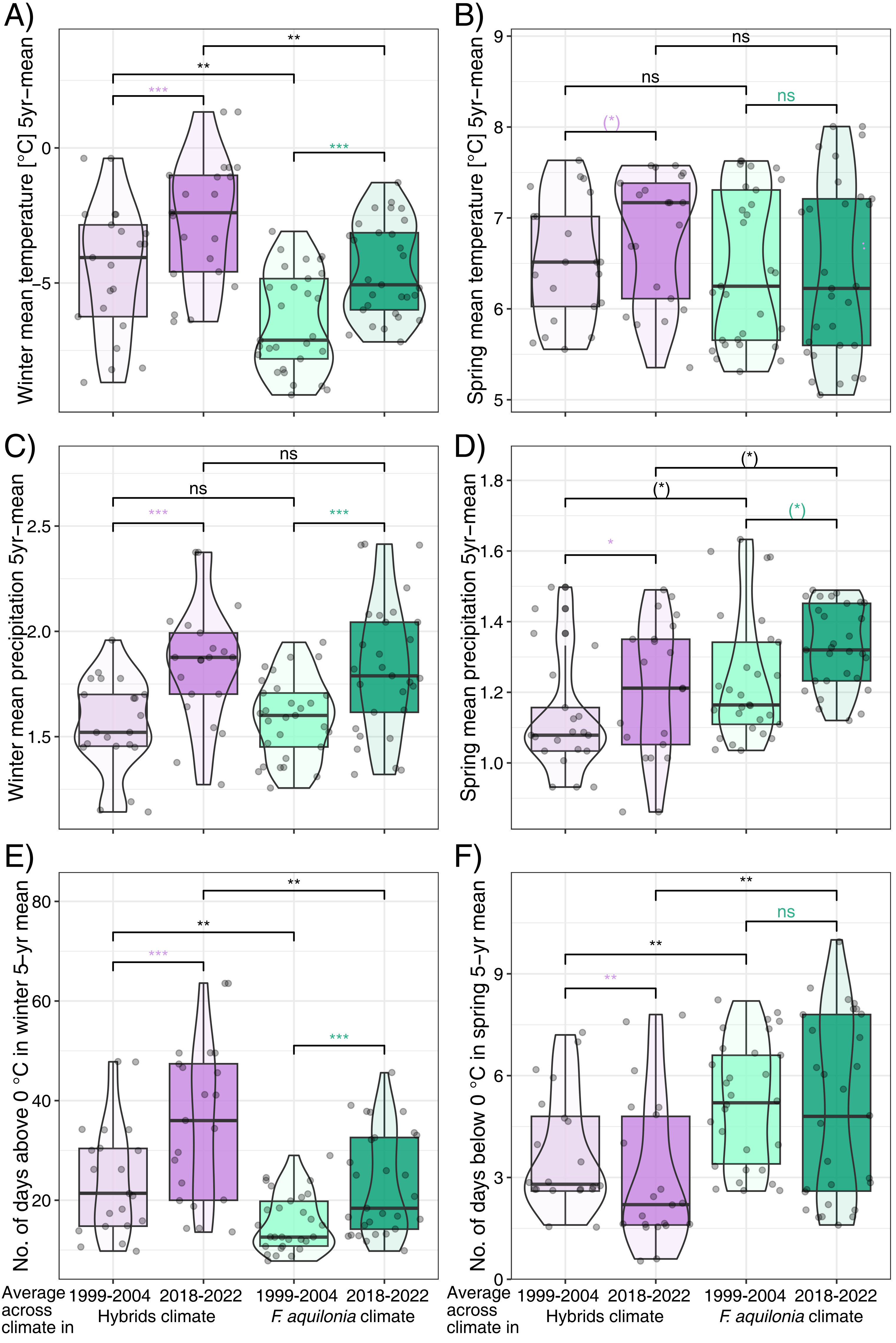
A)
Winter mean temperature [°C] 5yr−mean
B)
Spring mean temperature [°C] 5yr−mean
C)
Winter mean precipitation 5yr−mean
D)
Spring mean precipitation 5yr−mean
E)
No. of days above 0 °C in winter 5−yr mean
F)
No. of days below 0 °C in spring 5−yr mean
Average across climate in
1999-2004
2018-2022
1999-2004
2018-2022
Hybrids climate
F. aquilonia climate

Fig. S6. Environmental variables averaged over five years before the years 2005 (1999-2004) and 2023 (2018-2022) for the same locations. (A) Winter mean temperature, (B) spring mean temperature, (C) winter mean precipitation, (D) spring mean precipitation, (E) number of days above 0 °C in winter, (F) number of days below 0 °C in spring. Purple and green colours represent hybrids and *F. aquilonia*, respectively. Mean values were compared using Student's t-tests. Comparisons within hybrids are coloured with purple asterisks and within *F. aquilonia* with green asterisks. Parentheses around asterisks indicate non-significance after correction for multiple testing with * <0.05, ** <0.01, and ***<0.001. Sample numbers $N_{hybrids} = 21$ and $N_{F.\ aquilonia} = 29$. If the climate has changed over 18 years, we expect to observe similar climatic changes in the sympatric area in both hybrid and *F. aquilonia* populations in key climatic variables. The winter of the years 2018-2022 was warmer, had more precipitation, and more days above 0 °C than the winter of the years 1999-2004 in both hybrid and *F. aquilonia* populations (coloured asterisks). Also, the spring of 2018-2022 had more precipitation and fewer frost days below 0 °C in hybrid but not *F. aquilonia* populations than the spring of the years 1999-2004 (coloured asterisks). Additionally, in hybrid populations, the winter was warmer and had more days above 0 °C, while the spring had fewer frost days below 0 °C compared to *F. aquilonia* populations in both 5-year average periods (all other variables did not differ between years and/or populations). The interquartile range (25th-75th percentiles) is represented by the box, while the median is represented by the line within the box. Most extreme observations within the 1.5 times the interquartile range below and above the percentiles are presented with whiskers. Observations beyond the whiskers are presented as dots. Related to Figure 2.

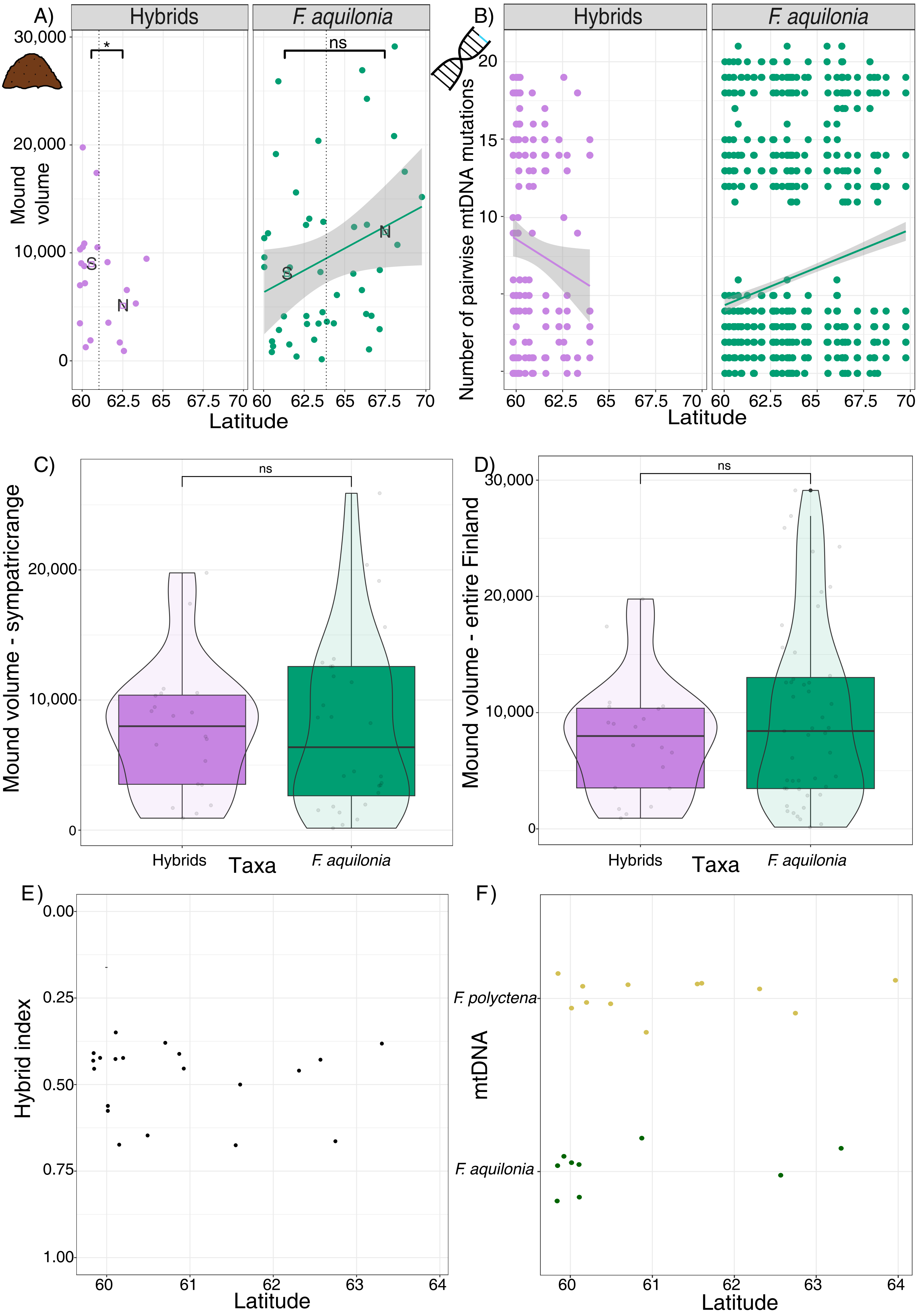

A)
Hybrids
F. aquilonia
30,000
20,000
10,000
0
Mound volume
*
ns
S
N
60
62.5
65
67.5
70
Latitude
B)
Hybrids
F. aquilonia
20
15
10
Number of pairwise mtDNA mutations
Latitude
C)
ns
Mound volume - sympatricrange
Hybrids
Taxa
F. aquilonia
D)
ns
Mound volume - entire Finland
E)
0.00
0.25
0.50
0.75
1.00
Hybrid index
59
60
61
62
63
64
F)
F. polyctena
F. aquilonia
mtDNA

Fig. S7. Mound volume, pairwise mutations, hybrid index, and mtDNA origin plotted against latitude. (A) Calculated mound volume (based on mound length, width, and height) plotted against latitude for hybrids (N=21) and *F. aquilonia* (N=48). The dotted lines represent the latitudinal means for both hybrids and *F. aquilonia*. They split the data in a 'Northern' and 'Southern' area used to compare the means, which are represented as squares. Squares with 'S' and 'N' denote the means for the ''Southern' and 'Northern' area, respectively. Statistical significance was calculated using one-sided t-tests with p-value * <0.05. (B) Number of pairwise mutations within hybrids and *F. aquilonia* separately plotted against the latitude for hybrids (N=572) and *F. aquilonia* (N=7310) using a linear model. Here, we combined data from the 'current' and 'previous' sampling to increase statistical power. (C) The mound volume did not differ between hybrids and *F. aquilonia* in the sympatric range (means, hybrids: 8993.60; *F. aquilonia*: 10,948.11; one-sided Mann-Whitney-U-test, W = 466, p-value = 0.6274) or (D) when tested across entire Finland (means, hybrids: 8993.60; *F. aquilonia*: 9892.90; one-sided Mann-Whitney-U-test, W = 312, p-value = 0.892). For C) and D), the interquartile range (25th-75th percentiles) is represented by the box, while the median is represented by the line within the box. Most extreme observations within the 1.5 times the interquartile range below and above the percentiles are presented with whiskers. Observations beyond the whiskers are presented as dots. We did not find a correlation between (E) hybrid index and latitude or (F) mtDNA origin (either *F. aquilonia* or *F. polyctena*) and latitude. Related to Figure 2.

Table S2. Number (and percentages) of assigned species and hybrid samples collected across Finland

Taxa	‘Previous‘ sampling 2005-2019	‘Previous’ and ‘current’ sampling - old and new populations	‘Current’ sampling from 2023 – revisited populations	‘Current’ sampling from 2023 – only new populations
F. aquilonia	45 (52%)	48 (52%)	22 (50%)	26 (54%)
F. lugubris	8 (9%)	11 (12%)	4 (9%)	7 (15%)
F. polyctena	6 (7%)	0 (0%)	0 (0%)	0 (0%)
F. pratensis	7 (8%)	3 (3%)	2 (5%)	1 (2%)
F. rufa	6 (7%)	6 (7%)	4 (9%)	2 (4%)
F. aquilonia × *F. polyctena* hybrids (‘Admixed1’ in (*28*)	12 (14%)	21 (23%)	11 (25%)	10 (21%)
F. aquilonia × *F. lugubris* × *F. rufa* × *F. pratensis* (‘Admixed2’ in (*28*)	3 (3%)	1 (1%)	1 (2%)	0 (0%)
F. lugubris × *F. polyctena/rufa*	0 (0%)	2[#] (2%)	0 (0%)	2 (4%)
**Total numbers**	**87**	**92**	**44**	**48**

Note: # represents a newly detected hybrid combination. The ‘previous’ sampling[37] was collected mainly in 2005-2008 with additional samples collected until 2019. The ‘current’ sampling uses samples collected in 2023. Related to Figure 1.

Table S3. Mean within-mound temperature values measured from 2020 to 2023 for each month and for hybrid and *F. aquilonia* populations

Month of the year	Mean values hybrids	Mean values *F. aquilonia*	Mann-Whitney-U W	Mann-Whitney-U p-value	p-value corrected for multiple testing
01	2.12	1.40	179903.0	<0.001	***
02	0.96	0.01	179534.5	<0.001	***
03	2.07	0.57	244460.5	<0.001	***
04	6.94	4.26	218912.5	<0.001	***
05	14.68	13.41	257571.5	<0.001	**
06	22.49	22.17	232133.5	0.218	ns
07	23.23	24.19	202922.5	<0.001	***
08	23.22	23.93	154003.5	<0.001	**
09	16.46	15.41	64365.0	0.001	**
10	12.44	11.45	13015.5	0.004	*
11	10.23	8.52	377.0	0.269	ns
12	5.38	4.58	45878.0	<0.001	***

Significant comparisons are represented with asterisks, where * denotes p-value < 0.05, ** denotes p-value < 0.01, and *** denotes p-value < 0.001. ns = not significant. Related to Figure 1.

Table S4. Summary of the proportional hazard ratios.

Variable	Coefficient	Standard error of the coefficient	Hazard ratio	95% CI	p-value
**Species - *F. aquilonia***	**1.04**	**0.47**	**2.82**	**1.11 - 7.13**	**0.028**
**mtDNA *F. polyctena***	**-0.60**	**0.27**	**0.55**	**0.32 – 0.93**	**0.027**
**Hybrid index**	**-0.79**	**0.26**	**0.45**	**0.27 – 0.77**	**0.003**
Max. temperature 21 days prior to sampling	-0.22	0.17	0.80	0.58 – 1.11	0.182
Min. temperature 21 days prior to sampling	0.10	0.14	1.11	0.94 – 1.46	0.477
Max. temperature 5 days prior to sampling	0.29	0.17	1.33	0.96 – 1.85	0.088
Temperature ranges 7 days prior to sampling	-0.10	0.11	0.91	0.73 – 1.23	0.382
Temperature ranges 3 days prior to sampling	0.21	0.11	1.23	0.99 – 1.53	0.066
**Max. precipitation 1 day prior to sampling**	**0.34**	**0.10**	**1.40**	**1.14 – 1.71**	**0.001**
**Precipitation ranges 7 days prior to sampling**	**0.31**	**0.13**	**0.73**	**0.57 – 0.95**	**0.018**

CI = Confidence intervals. Bold values represent statistically significant values. Related to Figure 1.

Table S5. Survival probability at measured temperatures and time points in the heat assay of hybrid and *F. aquilonia* populations in the sympatric area (i.e. overlapping geographic) and entire Finland

**Area**	**Temperature [°C] / time point when measured**	**Hybrid survival probability ± confidence intervals**	***F. aquilonia* survival probability ± confidence intervals**	**Two-sample proportion test**
**Sympatric area**	42.4 °C / 8 min	1.00 ± 1.00-1.00	0.96 ± 0.92-1.00	$X^2 = 2.30$<br>df = 1<br>p-value = 0.065
	**44.0 °C / 9 min**	**0.99 ± 0.97-1.00**	**0.89 ± 0.83-0.95**	$\mathbf{X^2 = 7.18}$<br>**df = 1**<br>**p-value = 0.004**
	44.8 °C / 10 min	0.78 ± 0.69-0.87	0.71 ± 0.63-0.80	$X^2 = 0.95$<br>df = 1<br>p-value = 0.165
	44.7 °C / 11 min	0.57 ± 0.47-0.68	0.60 ± 0.51-0.69	$X^2 = 0.08$<br>df = 1<br>p-value = 0.613
**Entire Finland**	42.4 °C / 8 min	1.00 ± 1.00-1.00	0.97 ± 0.95-0.99	$X^2 = 1.35$<br>df = 1<br>p-value = 0.122
	**43.9 °C / 9 min**	**0.99 ± 0.97-1.00**	**0.89 ± 0.84-0.93**	$\mathbf{X^2 = 7.18}$<br>**df = 1**<br>**p-value = 0.004**
	44.8 °C / 10 min	0.78 ± 0.69-0.87	0.68 ± 0.62-0.75	$X^2 = 2.06$<br>df = 1<br>p-value = 0.08
	44.7 °C / 11 min	0.57 ± 0.47-0.68	0.57 ± 0.51-0.65	$X^2 < 0.01$<br>df = 1<br>p-value = 0.500

Note: We selected four time points and their respective temperatures close to 45 °C, which is the temperature when most ants fell into heat coma. Note: Bold values represent statistically significant values based on confidence interval differences. df = degrees of freedom. Related to Figure 2.

Table S6. Observed species turnover of same mound or mound in near surroundings

Taxa turnover	Previous -> Now	Number of populations
Formica species to hybrids	*F. rufa* -> *F. aquilonia* × *F. polyctena* hybrid	1
	F. lugubris -> Admixed2 Admixed2 is a *F. aquilonia* × *F. lugubris* × *F. rufa* × *F. pratensis* hybrid indicating that the previous assignment to *F. lugubris* was due to a limited sample size	1
	F. pratensis -> *F. aquilonia* × *F. polyctena* hybrid	1
	F. aquilonia -> *F. aquilonia* × *F. polyctena* hybrid	1
Admixed2 to hybrid	Admixed2 -> *F. aquilonia*× *F. polyctena* hybrid (exact same mound)	1
hybrid to *Formica* species	*F. aquilonia* × *F. polyctena* hybrid -> *F. rufa*	1
	F. aquilonia × *F. polyctena* hybrid -> *F. aquilonia*	2
Formica species to other *Formica* species	*F. aquilonia* -> *F. rufa* (exact same mounds)	2
	F. aquilonia -> *F. lugubris*	1
	F. lugubris -> *F. aquilonia* (exact same mound)	1

Note: In total, we detected 4 same mounds and 8 mounds in the surroundings with different species or hybrids. Related to Figure 2.

Table S7. Comparing 5-year climatic averages (mean and bootstrapped tests) across 'established and the 'expanded' ranges and between time

Variable	Comparisons p-value / bootstrapped p-value	'Established' range with hybrids *present* in 2005 - lower latitudes (n=5)	'Expanded range' with hybrids *absent* in 2005 - higher latitudes (n=4)
**Winter mean temperature**	**'Expanded range' with hybrids *absent* in 2005 - higher latitudes (n=4)**	0.150 / **0.006****	-
	**'Expanded range' with hybrids *present* in 2023 - higher latitudes (n=4)**	0.730 / 0.442	0.230 / **0.006****
**Spring mean temperature**	**'Expanded range' with hybrids *absent* in 2005 - higher latitudes (n=4)**	0.098 / **0.004****	-
	**'Expanded range' with hybrids *present* in 2023 - higher latitudes (n=4)**	**0.048** / **<0.001*****	0.886 / 0.840
**Winter mean precipitation**	**'Expanded range' with hybrids *absent* in 2005 - higher latitudes (n=4)**	1.00 / 0.792	-
	**'Expanded range' with hybrids *present* in 2023 - higher latitudes (n=4)**	1.00 / 0.916	1.00 / 0.792
**Spring mean precipitation**	**'Expanded range' with hybrids *absent* in 2005 - higher latitudes (n=4)**	0.260 / **0.012***	-

	**‘Expanded range’ with hybrids *present* in 2023 - higher latitudes (n=4)**	0.260 / **0.024***	0.690 / 0.666
**Winter number of days above 0 °C**	**‘Expanded range’ with hybrids *absent* in 2005 - higher latitudes (n=4)**	0.150 / **0.006****	-
	**‘Expanded range’ with hybrids *present* in 2023 - higher latitudes (n=4)**	0.340 / 0.130	0.340 / **0.032***
**Spring number of days below 0 °C**	**‘Expanded range’ with hybrids *absent* in 2005 - higher latitudes (n=4)**	0.120 / **0.004****	-
	**‘Expanded range’ with hybrids *present* in 2023 - higher latitudes (n=4)**	0.110 / **<0.001*****	0.340 / 0.572

Note: Parentheses around a p-value indicate its non-significance after correction for multiple testing. Note, we use the terms ‘2023’ and ‘2005’ for simplicity but refer to the 5-year climatic average of the years 2018-2022 and 1999-2004, respectively. Significant comparisons are represented with asterisks, where * denotes p-value < 0.05, ** denotes p-value < 0.01, and *** denotes p-value < 0.001. Related to Figure 2.

Table S8. Comparing 5-year climatic averages between hybrid and *F. aquilonia* populations

Variable	Year (5-year average)	Variable mean hybrids	Variable mean *F. aquilonia*	Test, test statistic, (df), p-value
**Winter mean temperature**	2005 (1999-2004)	-4.50	-6.45	**t-test, t = -3.09, df = 35.52, p-value = 0.004**
	2023 (2018-2022)	-2.63	-4.53	**t-test, t = -3.14, df = 35.38, p-value = 0.004**
**Test, test statistic, df, p-value**		**paired t-test, t = -49.44, df = 20, p-value<0.001**	**paired t-test, t = -64.39, df = 28, p-value<0.001**	
**Spring mean temperature**	2005 (1999-2004)	6.53	6.45	Wilcox-test, W = 276, p-value = 0.582
	2023 (2018-2022)	6.80	6.42	Wilcox-test, W = 225, p-value = 0.120
**Test, test statistic, df, p-value**		paired Wilcox-test, V = 55, (p-value = 0.037)	paired Wilcox-test, V = 253, p-value = 0.449	
**Winter mean precipitation**	2005 (1999-2004)	1.56	1.59	t-test, t = 0.48, df = 39.10, p-value = 0.638
	2023 (2018-2022)	1.84	1.83	t-test, t = -0.17, df = 45.85, p-value = 0.866
**Test, test statistic, df, p-value**		**paired t-test, t = -6.43, df = 20, p-value<0.001**	**paired t-test, t = -5.35, df = 28, p-value<0.001**	

**Spring mean precipitation**	2005 (1999-2004)	1.13	1.24	**Wilcox-test, W = 434, p-value = 0.011**
	2023 (2018-2022)	1.21	1.33	Wilcox-test, W = 411, (p-value = 0.037)
**Test, test statistic, df, p-value**		paired Wilcox-test, V = 54, (p-value = 0.034)	paired Wilcox-test, V = 117, (p-value = 0.031)	
**Winter number of days above 0 °C**	2005 (1999-2004)	24.27	15.50	**Wilcox-test, W = 156, p-value = 0.004**
	2023 (2018-2022)	35.74	23.12	**Wilcox-test, W = 149.5, p-value = 0.002**
**Test, test statistic, df, p-value**		**paired Wilcox-test, V = 0, p-value<0.001**	**paired Wilcox-test, V = 0, p-value<0.001**	
**Spring number of days below 0 °C**	2005 (1999-2004)	3.73	5.21	**Wilcox-test, W = 446.5, p-value = 0.005**
	2023 (2018-2022)	3.05	5.00	**Wilcox-test, W = 456.5, p-value = 0.003**
**Test, test statistic, df, p-value**		**paired Wilcox-test, V = 205, p-value = 0.002**	paired Wilcox-test, V = 261.5, p-value = 0.347	

Mean values and statistical results are denoted for the six environmental variables. Note: Parentheses around a p-value indicates its non-significance after correction for multiple testing. Significant values are in bold font. df = degrees of freedom. Related to Figure 2.

Data S1. (separate file) Data1.xlsx. Excel file with all relevant data to re-analyse the main results.

Data S2. (separate file) Wood_ants_Finland_MS.R. R-script to re-analyse the main results.
